# Epigenetic regulation of puberty via Zinc finger protein-mediated transcriptional repression

**DOI:** 10.1038/ncomms10195

**Published:** 2015-12-16

**Authors:** Alejandro Lomniczi, Hollis Wright, Juan Manuel Castellano, Valerie Matagne, Carlos A. Toro, Suresh Ramaswamy, Tony M. Plant, Sergio R. Ojeda

**Affiliations:** 1 Division of Neuroscience, Oregon National Primate Research Center, Beaverton, Oregon 97006, USA; 2 Department of Cell Biology, Physiology and Immunology, University of Cordoba; CIBER Fisiopatología de la Obesidad y Nutrición, Instituto de Salud Carlos III; and Instituto Maimónides de Investigación Biomédica (IMIBIC)/Hospital Universitario Reina Sofia (HURS), Cordoba 14004, Spain; 3 Department of Obstetrics, Gynecology and Reproductive Sciences, University of Pittsburgh School of Medicine, Pittsburgh, Pennsylvania 15261, USA

## Abstract

In primates, puberty is unleashed by increased GnRH release from the hypothalamus following an interval of juvenile quiescence. GWAS implicates *Zinc finger* (*ZNF*) genes in timing human puberty. Here we show that hypothalamic expression of several *ZNF*s decreased in agonadal male monkeys in association with the pubertal reactivation of gonadotropin secretion. Expression of two of these *ZNF*s, *GATAD1* and *ZNF573*, also decreases in peripubertal female monkeys. However, only *GATAD1* abundance increases when gonadotropin secretion is suppressed during late infancy. Targeted delivery of *GATAD1* or *ZNF573* to the rat hypothalamus delays puberty by impairing the transition of a transcriptional network from an immature repressive epigenetic configuration to one of activation. GATAD1 represses transcription of two key puberty-related genes, *KISS1* and *TAC3*, directly, and reduces the activating histone mark H3K4me2 at each promoter via recruitment of histone demethylase KDM1A. We conclude that GATAD1 epitomizes a subset of ZNFs involved in epigenetic repression of primate puberty.

## Abstract (web summary)

Zinc finger (ZNF) genes are implicated in timing human puberty. Here, the authors show that GATAD1, a ZNF protein, represses transcription of key puberty-activating genes by recruiting histone demethylase KDM1A to their promoters, suggesting GATAD1 epitomizes a subset of ZNFs involved in repression of primate puberty.

## Graphical abstract

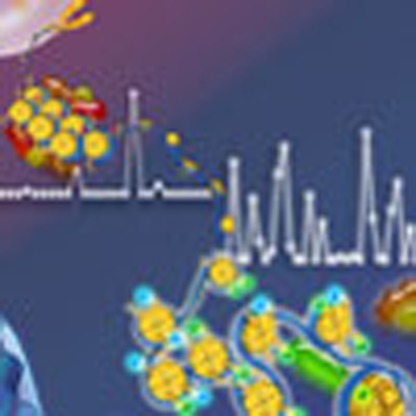

Puberty in highly evolved primates is unleashed after a prolonged phase of juvenile development by the re-emergence of a robust pattern of pulsatile GnRH release, a mode of hypothalamic neuroendocrine activity that was placed in check during infancy^1^. One immediate cause of this juvenile hiatus in GnRH release appears to be a reduced production of the peptide kisspeptin from a subset of *KISS1* neurons that are located in the arcuate nucleus (ARC) of the medial basal hypothalamus (MBH). Because many of these *KISS1* neurons also express neurokinin B and dynorphin, they have been termed KNDy neurons^2^,^3^. Both the reduced activity of the GnRH pulse generator during juvenile development and the resurgence of GnRH secretion at puberty occur in the absence of the gonads, indicating that these changes do not require feedback actions of either ovarian or testicular steroids in primates^1^.

One of our laboratories recently provided evidence that a mechanism of transcriptional repression prevents the premature initiation of puberty in the female rat by silencing the *Kiss1* gene in KNDy neurons^4^. Transcriptional repression is a major component of the molecular machinery regulating mammalian gene expression. Of the 2,000 transcription factors estimated to be present in the human genome^5^, almost 800 are transcriptional repressors containing zinc finger (ZNF) motifs^6^. These motifs recognize specific DNA sequences in regulatory regions of the genome^7^, and therefore are critical for ZNF proteins to interact with DNA targets and regulate gene expression. The most common of these motifs is the C2H2 or Krüppel type of ZNF (KZNF)^6^. About one third of KZNFs also contain a motif called a Krüppel-associated box (KRAB) that recruits histone deacetylase complexes to the DNA region to which the ZNF is attached^8^.

The potential contribution of *ZNF* genes to the pubertal process was recently suggested by several genome-wide association studies (GWAS) showing that single-nucleotide polymorphisms located near ZNF131, ZNF462 and ZNF483 were associated with an earlier age of menarche^9^,^10^,^11^. These observations raise the possibility that members of the *ZNF* gene superfamily, operating within the neuroendocrine brain, may represent a component of the neurobiological brake that underlies the arrest of the GnRH pulse generator during juvenile development in primates and thus contribute to preventing the premature initiation of puberty.

To systematically pursue this idea, we used DNA arrays to interrogate the hypothalamic transcriptome of agonadal male rhesus monkeys at two critical phases of postnatal development, the infantile–juvenile (INF–JUV) transition when the GnRH pulse generator is brought into check and the juvenile–pubertal (JUV–PUB) transition when robust GnRH pulse generator activity is re-initiated. We employed agonadal male monkeys to eliminate the confounding effect of gonadal steroids on gene expression in the brain, and because the juvenile hiatus in GnRH release is more conspicuous than that in females^1^. We also determined if any of the changes observed in males also occurred in females. To gain mechanistic insights into the actions of ZNFs, we used female rats as an *in vivo* model of puberty timing, and a combination of targeted DNA arrays, systems biology and *in vitro* molecular approaches. Our results suggest that ZNFs restrain puberty by epigenetically repressing a gene network that—operating in the ARC—controls puberty by governing pulsatile GnRH release.

## Results

### Gonadotropin levels in agonadal male monkeys

**Figure 1:**
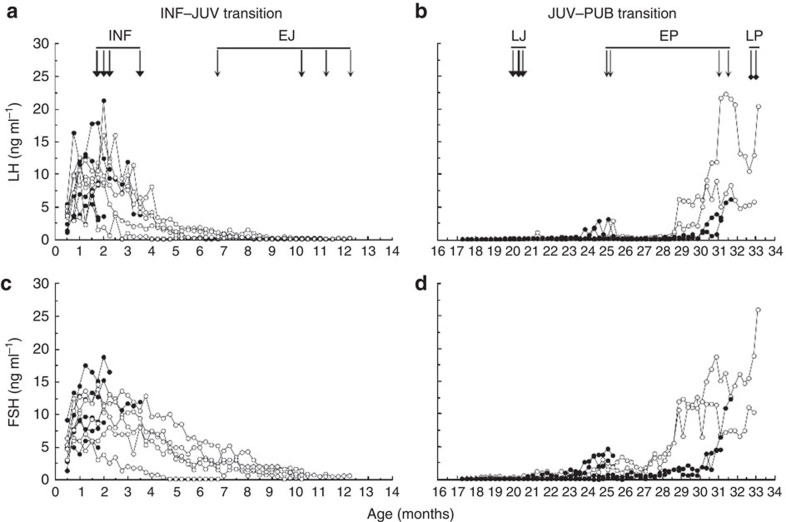
Patterns of gonadotropin secretion in agonadal male monkeys. (**a**,**c**) Infant (INF)–juvenile (JUV) transition. Time courses of circulating concentrations of LH (**a**) and FSH (**c**) in 10 individual monkeys bilaterally orchidectomized at 2–6 days of age. Data for animals comprising the infantile (INF; *n*=5) and early juvenile (EJ; *n*=5) groups are shown by the closed and open data points, respectively. Block and line arrowheads indicate age at which the hypothalamus was collected from INF and EJ animals, respectively. (**b**,**d**) Juvenile (JUV)–pubertal (PUB) transition. Time courses of circulating concentrations of LH (**b**) and FSH (**d**) in 10 individual monkeys bilaterally orchidectomized at 16–18 months of age. Data for animals comprising the early pubertal (EP; *N*=4) and late pubertal (LP; *n*=2) groups are shown by the closed and open data points, respectively. Data for late juveniles (LJ) (*n*=4) are shown in open triangles—note these are largely masked by the closed data points. Block, line and square arrowheads indicate age at which the hypothalamus was collected from LJ, EP and LP animals, respectively. Thicker arrows are the result of two animals with overlapping ages.

Consistent with earlier observations^1^, when monkeys were orchidectomized shortly after birth, serum luteinizing hormone (LH) and follicle stimulating hormone (FSH) levels (reflecting pulsatile GnRH release) increased immediately and remained elevated for 3–4 months before decreasing to reach low, often undetectable, values by 9–12 months of age (Fig. 1a,c). Similarly, when animals were orchidectomized at the mid-juvenile stage (16 months of age), serum LH and FSH levels remained low for at least 7–8 months, before increasing towards pubertal values (Fig. 1b,d; see also Supplementary Information).

### ZNF expression during the JUV–PUB transition

**Figure 2:**
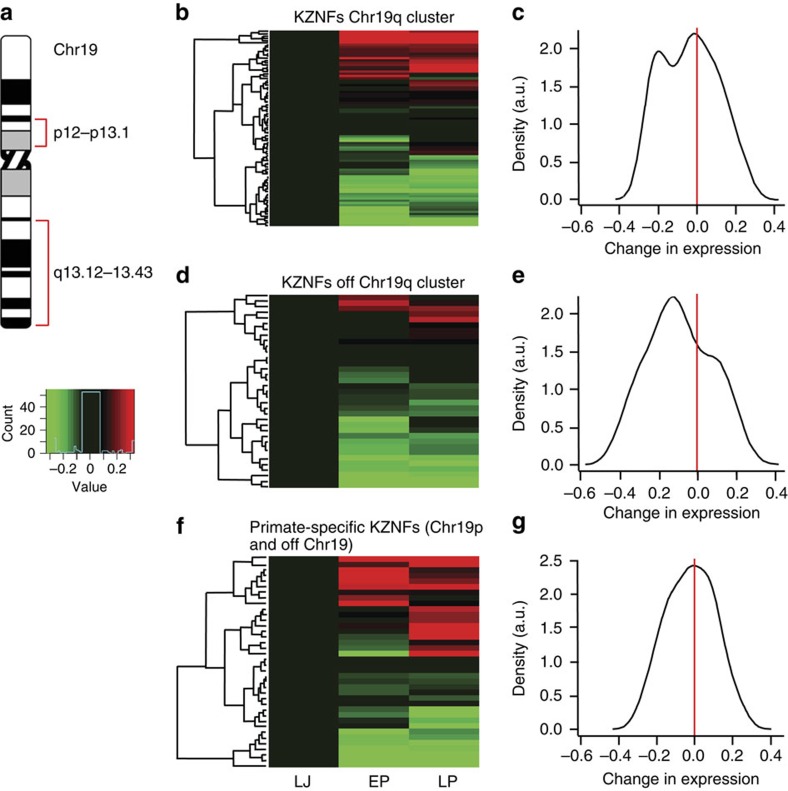
Heat maps of ZNF expression in the MBH of agonadal monkeys. (**a**) Schematic representation of human chromosome 19 illustrating the location of two clusters of *KZNF* genes, the largest in the q arm (q13.12–13.43 region) and another consisting of primate-specific genes in the p arm (p12–p13.1 region). (**b**) Heat map illustrating the changes in expression of the KZNF cluster located in the q arm during the juvenile-pubertal transition. (**c**) Kernel density plot of the changes shown in **b**. (**d**) Heat map illustrating the changes in expression of KZNFs located in chromosomes other than chromosome 19 during the juvenile–pubertal transition. (**e**) Kernel density plot of the changes shown in **d**. (**f**) Heat map of changes in expression of the primate-specific ZNF cluster located in either the p arm of chromosome 19 or chromosomes other than chromosome 19. (**g**) Kernel density plot of the changes shown in **f**. LJ (*n*=4), EP (*n*=4), LP (*n*=2).

Using Affymetrix GeneChip Rhesus macaque Genome Arrays to interrogate the MBH of agonadal male monkeys, we observed a significant (*P*=0.0065; hypergeometric test) enrichment in the category of transcriptional regulation within a group of 822 genes with decreased expression at the time of the pubertal rise in LH secretion (Supplementary Data 1). A conspicuous fraction of these genes belongs to the ZNF family of transcriptional repressors. Heat maps constructed using 161 ZNFs (Supplementary Data 2) with present calls in the array, revealed that 89 ZNFs belonging to the large *KZNF* gene cluster located on chromosome 19q (Fig. 2a; Supplementary Data 2), and 32 KZNFs located in chromosomes other than Ch19 (Supplementary Data 2), had a collective expression profile with the advent of the pubertal LH rise left-skewed towards decreased expression (Fig. 2b–e). This skewness was not observed in a set of 39 primate-specific ZNFs (Supplementary Data 2) mostly located in chromosomal region 19p12–p13.1 (Fig. 2a). Primate-specific genes were expressed in a symmetric, unimodal distribution with zero skewness (Fig. 2f,g), suggesting that they do not contribute to regulating the timing of puberty.

**Figure 3:**
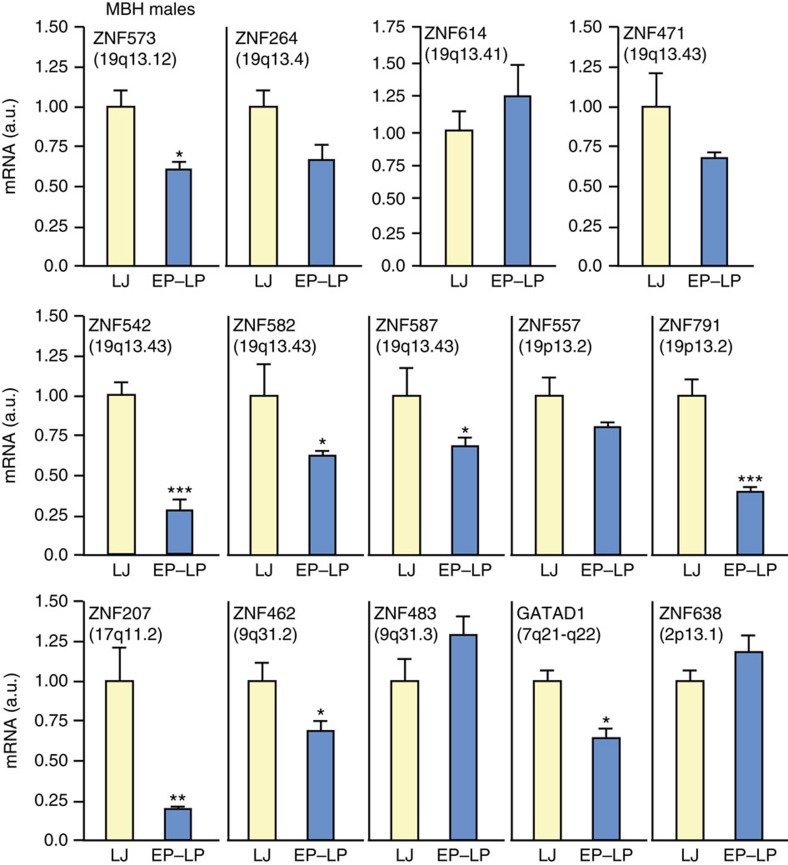
ZNF mRNA levels in the monkey MBH at the juvenile-pubertal transition. During the juvenile–pubertal transition low levels of circulating gonadotropins begin to increase reflecting the reactivation of GnRH pulse generation that triggers puberty in intact monkeys (Fig. 1). All animals were orchidectomized at 16–18 months of age. Because mRNA levels did not change further in LP as compared with EP these two groups were combined before statistical analysis. Vertical bars are means±s.e.m. LJ=late juvenile (*n*=4–6); EP=early puberty and LP=late puberty (*n*=5–8; *P<0.05, **P<0.01 and ***P<0.001 versus LJ group; Student's *t*-test).

Based on these observations we selected 14 KZNFs for quantitative PCR (qPCR) analysis and found that expression of eight of them significantly decreased at the time of the pubertal gonadotropin rise (Fig. 3). Five of these genes (*ZNF573*, *ZNF791*, *ZNF542/383*, *ZNF582* and *ZNF587*, listed in centromeric to telomeric order) are KRAB–KZNFs located on chromosomal region 19q13. However, not all KRAB–KZNFs showed a puberty-related expression profile, as the hypothalamic mRNA levels of *ZNF483*, a KRAB–KZNF located on a different chromosome (9q31.2–31.3) and implicated by GWAS in the timing of human menarche^9^,^10^, did not change at the time of the pubertal gonadotropin rise (Fig. 3).

Conversely, chromosome 19-located ZNFs were not the only ones exhibiting decreased expression in the hypothalamus at the time of the pubertal gonadotropin rise, as mRNA levels of three ZNFs lacking a KRAB domain and located in chromosomes other than chromosome 19 (*ZNF207*, *ZNF462* and *GATAD1*) decreased at this time (Fig. 3). *ZNF462* is located in the same chromosomal region as *ZNF483* and has also been implicated by GWAS as affecting the timing of menarche in girls^9^,^10^. *GATAD1* (GATA ZNF domain-containing protein 1, also known as *ODAG*, ocular development-associated gene) contains a ZNF domain at the N terminus^12^, and is located on chromosomal region 7q21–q22 (ref. 13). GWAS have implicated *GATAD1* as a gene associated with adult height^14^,^15^. GATAD1 is considered as a histone ‘reader' that recognizes promoter regions containing histone 3 trimethylated at lysine 4 (H3K4me3) to form a repressive complex^16^.

Of the eight ZNFs with decreased pubertal expression in the MBH of agonadal male monkeys, expression of five of them also decreased in the cerebral cortex (CTX) (Supplementary Fig. 1) suggesting that this developmental decrease was not hypothalamic specific. In contrast, mRNA levels of three ZNFs (*ZNF573*, *ZNF542* and *GATAD1*) remained unchanged in the CTX (Supplementary Fig. 1). To determine whether peripubertal repression of these three genes was associated with changes in expression of other genes previously implicated in the control of puberty (for example, *KISS1*, *TAC3*, *GnIH* and so on), we further mined the DNA array results and observed that expression of only *KISS1* and *TAC3* appeared to increase at the time when *ZNF573*, *ZNF542* and *GATAD1* expression decreased (Supplementary Data 3). QPCR analysis demonstrated that indeed both *KISS1* and *TAC3* mRNA content increased significantly in the MBH at this time (Supplementary Fig. 2). Of the three ZNFs showing a hypothalamic-specific decreased expression in males, only *GATAD1* and *ZNF573* behaved similarly in intact female monkeys of pubertal age (Supplementary Fig. 3), suggesting that these genes may be involved in the pubertal process of both sexes.

### ZNF expression during the INF–JUV transition

**Figure 4:**
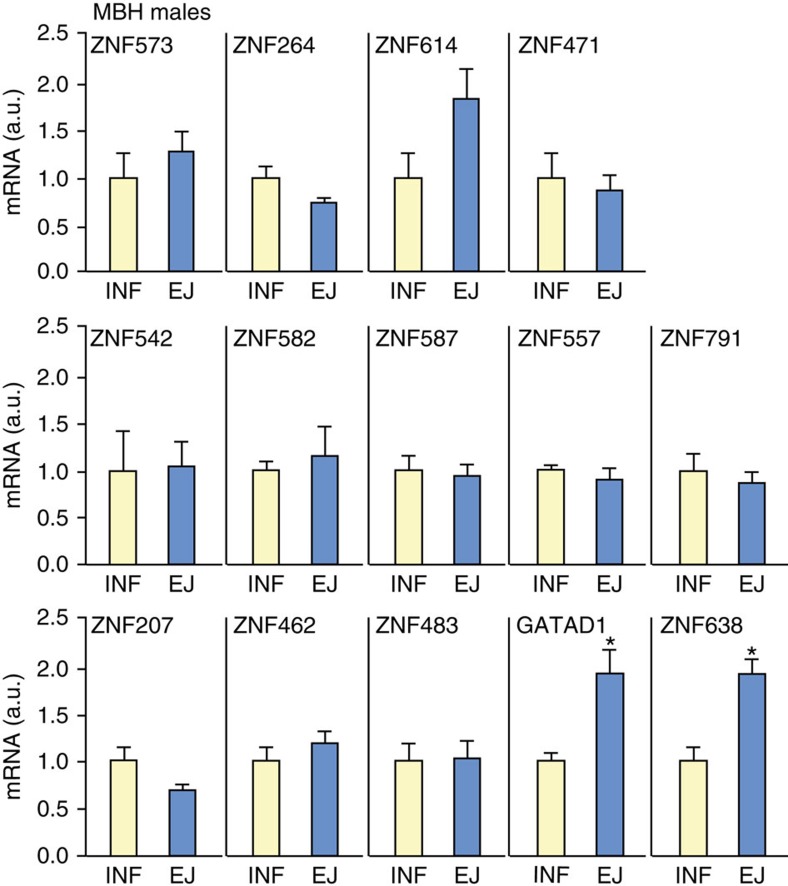
ZNF mRNA levels in the monkey MBH at the infantile–early juvenile transition. During the infantile–early juvenile transition, the elevated gonadotropin levels seen in infancy (reflecting a robust GnRH pulsatility) are brought into check resulting in the hypogonadotropic state of the juvenile phase of development. All animals were orchidectomized 2–6 days after birth. Vertical bars are means±s.e.m. INF=infant (*n*=3–5); EJ=early juvenile (*n*=4–5). (*=*P*<0.05 versus INF group; Student's *t*-test).

Of the eight ZNFs with decreased expression at the time of the pubertal reactivation of gonadotropin release in agonadal males, only *GATAD1* expression increased in the MBH at the time of the INF–JUV transition (Fig. 4). This finding suggests that GATAD1 may play a dual role in both silencing the GnRH pulse generator at the INF–JUV transition and the awakening of GnRH release at the onset of puberty.

To determine whether upregulation of *GATAD1* expression at this stage of postnatal development could be related to changes in expression of genes thought to be involved in the reactivation of GnRH pulse generator activity at the onset of puberty, we examined the DNA array results for a number of these genes including those associated with initiation (for example, *KISS1*, *TAC3* and so on), and delay (for example, *GnIH*, genes encoding opioid peptides, GABA-related peptides and so on) of puberty. None exhibited significant changes in the expression (Supplementary Data 4, Supplementary Fig. 2), indicating the need for additional research to unveil the mechanisms underlying GnRH pulse generation by the infant hypothalamus.

### *GATAD1* and *ZNF573* delay puberty in a rodent model

**Figure 5:**
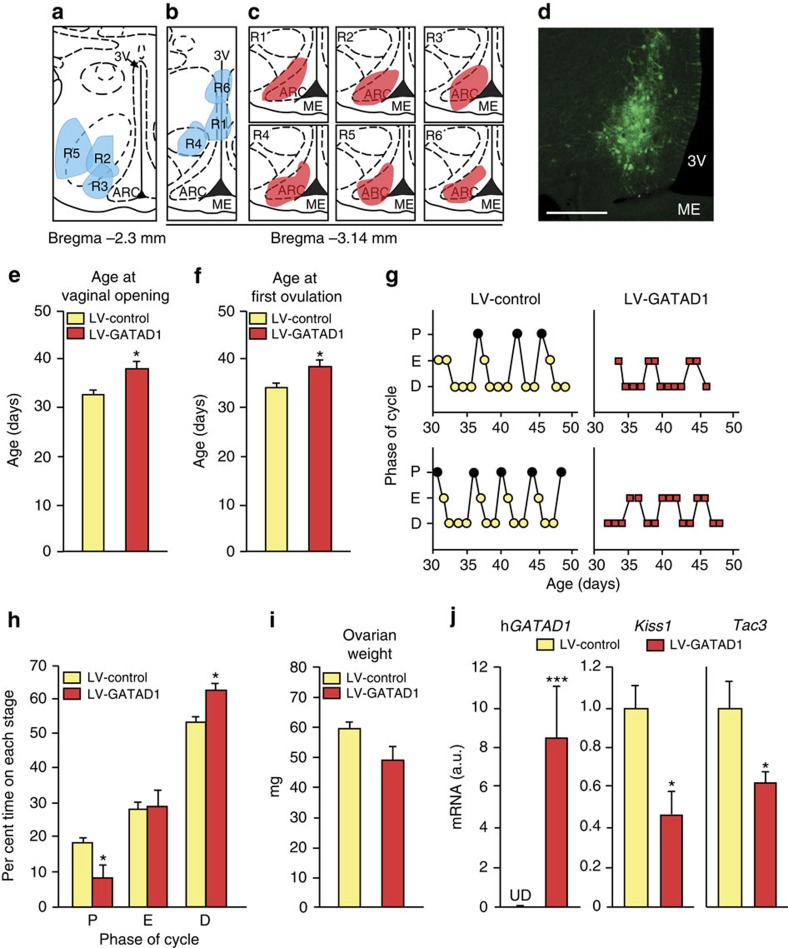
Overexpression of hGATAD1 in the ARC of immature female rats. (**a**–**c**) Diagrams showing the approximate location of microinjections intended to deliver a lentivirus construct expressing hGATAD1 (*LV-GATAD1*) to the ARC nucleus of prepubertal female rats. (**a**) Injections located anterior and lateral to the intended target (*n*=3); (**b**) injections located dorsal to the intended target (*n*=3); (**c**) injections correctly located in the body of the ARC (*n*=6). (**d**) Immunohistofluorescent localization of GFP-expressing cells in the ARC of a female rat 30 days after receiving injections of *LV-GATAD1*. The construct was stereotaxically delivered to the ARC on PND21. One coronal plane, showing the right side of the MBH is depicted. Bar, 200 μm. Because rats injected with an *LV-GFP* construct or rats with misplaced *LV-GATAD1* injections (too high or too rostral/lateral) behave similarly, they were combined into a single group (LV-control) for statistical purposes. (**e**) The age at vaginal opening was delayed in *LV-GATAD1*-injected rats. (*P<0.05 versus LV-control group; Student's *t*-Test) (**f**) The age of first ovulation, estimated by a predominance of leukocytes in vaginal lavages following an abundance of cornified cells (indicative of the first oestrus) was also delayed. (*P<0.05 versus LV-control group; Student's *t*-test; **g**) Examples of disrupted oestrous cyclicity in *LV-GATAD1*-injected rats. (**h**) Per cent of time spent in different stages of the oestrous cycles by rats injected with *LV-GATAD1* in the ARC (*LV-GATAD1*) versus control animals (LV-control). (*P<0.05 versus LV-control group; Student's *t*-test) (**i**) Ovarian weight in *LV-GFP* and *LV-GATAD1*-injected rats. (**j**) *hGATAD1*, *Kiss1* and *Tac3* mRNA levels detected by qPCR in the ARC of 28-day-old female rats receiving an *LV-GATAD1* (*n*=7) injection targeting the ARC on PND21. (*P<0.05 and ***P<0.001 versus LV-control group (*n*=8); Student's *t*-test). Vertical bars are means±s.e.m. ME=median eminence; 3 V=third ventricle. P=proestrous; E=oestrous; D=diestrous.

To determine whether modifying *GATAD1* or *ZNF573* expression alters the timing of puberty, we chose a gain-of-function approach. We cloned the coding region of h*GATAD1* tagged with the human influenza haematogglutinin epitope (HA) or human *ZNF573*-tagged with both MYC and FLAG epitopes into a lentivirus vector (LV) that expressed GFP (Supplementary Fig. 4a). Both constructs (termed *LV-GATAD1* and *LV-ZNF573*) express the proteins of interest in 293T cells (Supplementary Fig. 4b). Although endogenous *Gatad1* mRNA levels do not decrease in the MBH of female rats during prepubertal development (Supplementary Fig. 5) and *Znf573* has not been described in rats, we nevertheless used the immature female rat as an *in vivo* system to broadly test the concept that ZNFs can affect the timing of puberty. We delivered these constructs bilaterally to the ARC via stereotaxic surgery into 21-day-old female rats. Female rats are a much better model than male rats to study puberty in rodents because the timing and completion of puberty can be precisely defined by detecting both the age at vaginal opening and first ovulation. The latter is determined indirectly by the presence of leukocytes in vaginal lavages the day after the animals were in the oestrus phase of the reproductive cycle (for additional details see Supplementary Information). Control animals were injected with a construct expressing only green fluorescent protein (GFP; LV-GFP). Immunohistofluorescence analysis of the sites of injection at the time of euthanasia (3 weeks after vaginal opening) using an antiGFP antibody demonstrated that the injections of LV-GATAD1 were located lateral and rostral to the ARC in three animals (Fig. 5a), dorsal to the ARC in three others (Fig. 5b), and well-placed in the body of this nucleus in six rats (Fig. 5c,d). Both the age at vaginal opening and the age at first ovulation were delayed (Fig. 5e,f), and oestrous cyclicity was disrupted (Fig. 5g). *LV-GATAD1*-injected rats spent fewer days in proestrous and more days in diestrous than control animals (Fig. 5h). Although ovarian weight (measured at the time of euthanasia) was lower in *LV-GATAD1*-injected animals, this difference did not reach statistical significance (Fig. 5i). Similar changes were observed in animals injected with *LV-ZNF573* (Supplementary Fig. 6). Eight days after *LV-GATAD1* injection into the ARC (*n*=8), h*GATAD1* mRNA levels were readily measurable, in contrast to LV-control-injected animals (*n*=8) in which h*GATAD1* transcripts were undetectable (Fig. 5j). The presence of h*GATAD1* mRNA was accompanied by a significant decrease in both *Kiss1* and *Tac3* mRNA abundance (Fig. 5j). These results suggest that a subgroup of ZNFs, epitomized by *GATAD1* and *ZNF573*, operates within the mammalian hypothalamus to prevent the premature activation of gonadal function.

### GATAD1 disrupts prepubertal reorganization of gene networks

To determine whether overexpressing GATAD1 in the ARC alters the connectivity of genes deemed to be important for pubertal development, we assembled a set of 224 genes (Supplementary Data 5) involved in diverse cellular functions, including trans-synaptic communication, glia-to-neuron communication, cell adhesiveness, transcriptional control and epigenetic regulation (Supplementary Fig. 7). We then generated custom-made TaqMan Open Array plates (Applied Biosystems) to interrogate the ARC transcriptome at three phases of prepubertal development (infantile-PND14, early juvenile (EJ)—PND21 and late juvenile (LJ)—PND28). The latter corresponds to the end of the juvenile period. We also examined the ARC 7 days after targeting GATAD1 to this region in PND21 rats. The TaqMan Open Array reliably detected 147 of the 224 genes (Supplementary Data 6). Failure to detect all 224 transcripts may be due to low amplification efficiency of computationally selected primers and to the low amount of reverse-transcribed material used in each microreaction (33 nl), which makes less certain the detection of low-abundance transcripts. To analyse the network configuration and connectivity of these genes, we used a compressive-sensing-based approach^17^, which infers gene networks by identifying strong co-expression links between genes and uses these links to estimate the potential influence of each of these genes within a given transcriptional programme (Supplementary Information).

**Figure 6:**
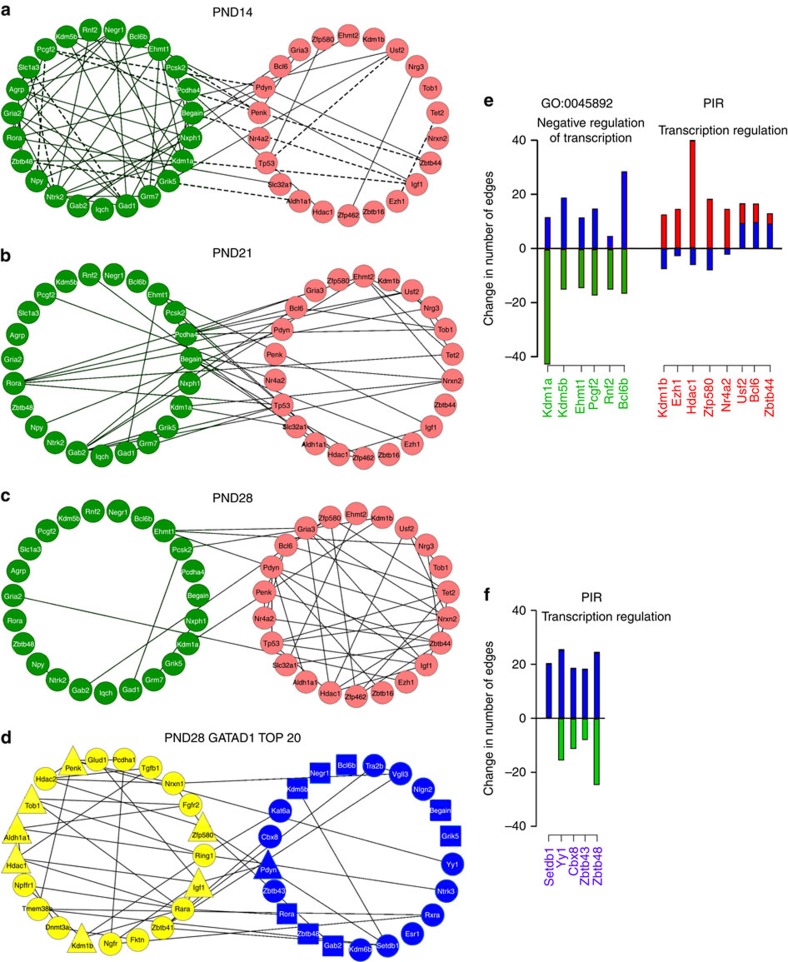
Effect of GATAD1 overexpression on gene co-expression networks. (**a**–**c**) Inferred co-expression network between the top 20 genes losing edges (green) and the top 20 genes gaining edges/connections (red) during prepubertal development, based on sum of changes in connectivity between PND14–PND21, PND14–PND28 and PND21–PND28. (**a**) Network at PND14. (**b**) Network at PND21. (**c**) Network at PND28. (**d**) Network at PND 28 with the top 20 genes losing edges (yellow) and gaining edges (blue) under GATAD1 overexpression as compared with the network inferred at PND28. In this network, genes that are among the top 20 genes gaining edges during normal prepubertal development are shown as triangles, while genes that are among the top 20 losers of connectivity during development are shown as squares. For all networks positive correlation edges are represented by solid black lines, negative correlation edges are shown as dashed lines. (**e**) Changes in connectivity of genes belonging to selected transcription-related functional categories that lose (Gene Ontology category GO:0045892: negative regulation of transcription, DNA-templated) or gain (Protein Information Resource, http://pir.georgetown.edu/; Keyword ‘transcription regulation') connectivity (green and red bars, respectively) from PND14 to PND28. For each gene the alteration in connectivity resulting from GATAD1 overexpresssion as compared with PND28 is shown as blue bars. (**f**) Genes encoding transcriptional repressors (annotated by ‘SP_PIR_KEYWORD' ‘transcription regulation') belong to the group of top 20 gainers of connectivity under GATAD1 overexpression (blue bars). Most of these genes (except *Setdb1*) showed decreased connectivity during normal prepubertal development (green bars).

Co-expression networks inferred using this method showed that the genes losing the most cumulative connectivity (edges) over time have considerable connectivity among themselves at PND14 (Fig. 6a, green circles), but they are also connected to gene sets that ultimately gain connectivity (Fig. 6a, red circles). By PND21, these relationships begin to shift (Fig. 6b), and by the JUV–PUB transition (PND28), the genes losing edges are almost entirely disconnected from each other (Fig. 6c, green circles), whereas genes gaining edges become more interconnected (Fig. 6c, red circles).

Overexpressing GATAD1 in the ARC at PND21 resulted in an immature pattern of connectivity at PND28 resembling that of PND14. The top 20 genes losing edges under GATAD1 overexpression, are normally richly interconnected at PND28 (Fig. 6d, yellow circles, triangles, squares; for definition of these shapes see legend to Fig. 6). Conversely, the top 20 genes gaining connectivity are poorly connected at PND28 (Fig. 6, blue circles, triangles, squares).

Both the top 20 losers and gainers of connectivity during prepubertal development were enriched for a number of functional annotations according to the DAVID functional enrichment analysis tool (Supplementary Data 7 and 8). Some of these functions would be expected to be important for the regulation of the pubertal process, because genes gaining edges between PND14 and PND28 are likely to have increased influence on the transcriptional programme of ARC cells as puberty approaches, while genes losing edges during the same time interval may become less influential. To determine whether GATAD1 overexpression changes the connectivity of these functionally annotated genes, we performed a computational analysis of data derived from two relevant functional enrichment categories, GO, using the category ‘negative regulation of transcription, DNA-templated', and the Protein Information Resource using the keyword ‘transcription regulation'. The results showed that epigenetic repressors, such as the histone demethylases *Kdm1a* and *Kdm5b*, and the PcG-related genes *Ehmt1*, *Pcgf2* and *Rnf2* lose connectivity as puberty approaches in the female rat (Fig. 6e, green bars), and that GATAD1 overexpression reverses this change (Fig. 6e, blue bars). We also identified at least three epigenetic regulators (*Kdm1b*, *Ezh1* and *Hdac1*), and one transcriptional repressor (*Zfp580*) among the top 20 genes gaining connectivity over normal development (Fig. 6e, red bars), but that fail to do so under GATAD1 overexpression (Fig. 6e, blue bars). Interestingly, several of the top 20 gainers of connectivity under GATAD1 overexpression (Fig. 6f, blue bars) were repressive genes, which normally either lose (*Yy1*, *Cbx8*, *Zbtb43* and *Zbtb48*) or maintain the same number of edges (*Setdb1*) between PND14 and PND28 (Fig. 6f, green bars). *Setdb1*, encodes an enzyme that trimethylates histone 3 at lysine 9 (ref. 18) (and therefore generates a repressive histone mark), *Yy1* and *Cbx8* are members of the PcG silencing complex, and *Zbtb43* and Zbtb48 are transcriptional repressors of the POZ-BTB family. Overall, these results strongly suggest that genes involved in epigenetic repression that lose influence in gene co-expression networks as puberty approaches regain this influence under GATAD1 overexpression.

### GATAD1 represses *KISS1* and *TAC3* transcription

**Figure 7:**
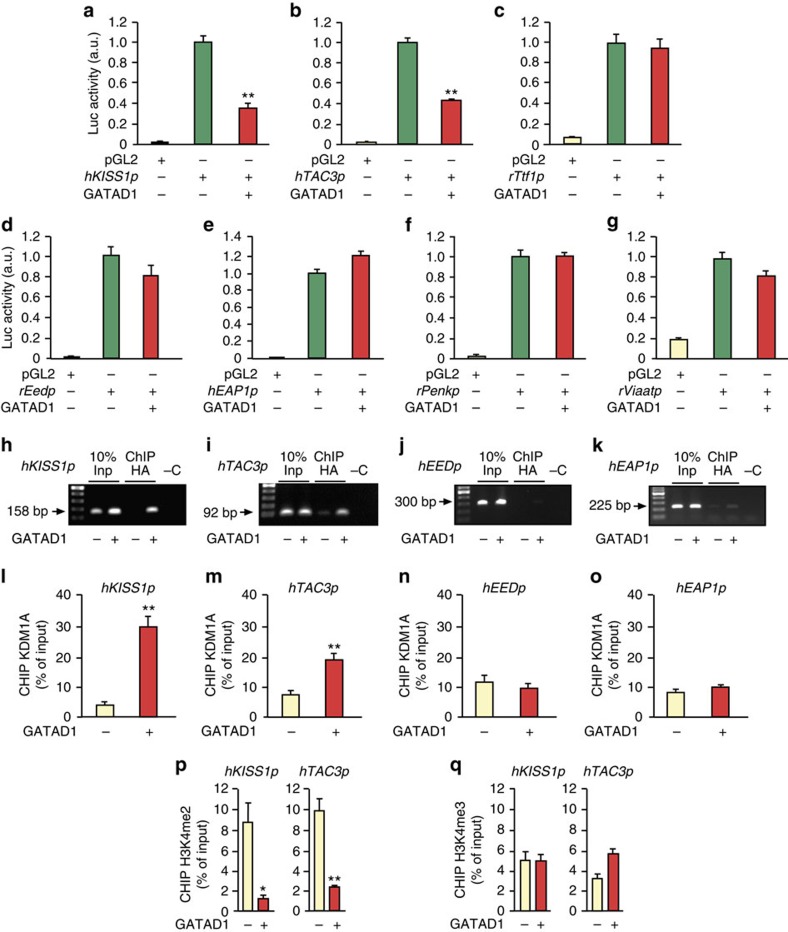
GATAD1 is a transcriptional repressor of the *KISS1* and *TAC3* genes. (**a**) GATAD1 represses h*KISS1* promoter activity as measured by gene reporter assays performed in Neuro2A cells; (**b**) GATAD1 also represses h*TAC3* promoter activity (**c**–**g**) GATAD1 does not alter the transcriptional activity of other puberty-related genes; (**c**) r*Tff1*, a transcriptional activator; (**d**) r*Eed*, a transcriptional repressor; (**e**) h*EAP1*, a transcriptional regulator with dual repressive/activating capabilities; (**f**) r*Penk*, a gene encoding encephalin a neuropeptide that inhibits GnRH secretion; (**g**) r*Viaat*, a gene encoding a vesicular inhibitory amino acid transporter, also considered to be inhibitory of the pubertal process (**P<0.01 versus GATAD1 (−) group (*n*=5); One-way ANOVA, Student–Newman–Keuls *post hoc* test). GATAD1 is recruited to (**h**) the h*KISS1* promoter, and (**i**) the h*TAC3* promoter, as assessed by ChIP assays performed in 293T cells; GATAD1 is not recruited to either (**j**) the h*EED* promoter, or (**k**) the h*EAP1* promoter; GATAD1 enhances recruitment of KDM1A/LSD1 to both (**l**) the h*KISS1* promoter, and (**m**) the h*TAC3* promoter; but not to the (**n**) h*EED* promoter or (**o**) h*EAP1* promoter. GATAD1 depletes H3K4me2 from the (**p**) h*KISS1* promoter and h*TAC3* promoter, without changing the abundance of (**q**) H3K4me3 (*P<0.05 and **P<0.01 versus GATAD1 (−) group (*n*=3); Student's *t*-test).

Promoter assays using constructs carrying the proximal promoter of several genes, including the puberty-activating genes *hKISS1*, *hTAC3* and *rTtf1*, and the puberty repressive genes *rEed*, *hEAP1*, *rpEnk* and r*Viaat* demonstrated that GATAD1 only repressed the promoter activity of *hKISS1* and *hTAC3* (Fig. 7a,b), without affecting the promoter activity of any of the other genes (Fig. 7c–g).

Chromatin immunoprecipitation (ChIP) assays showed strong association of GATAD1 to the proximal promoter of both h*KISS1* and h*TAC3* (Fig. 7h,i). Consistent with the results of promoter assays, no significant GATAD1 association to two other promoters tested, *hEED* and *hEAP1*, was detected (Fig. 7j,k).

The network analysis of the ARC showed that connectivity of *Kdm1a/*Lsd1 decreases immediately before puberty, and that this decrease was prevented by GATAD1 overexpression. Because KDM1A, the product of the *Kdm1a/*Lsd1 gene, selectively demethylates H3K4me2 at gene promoters^19^, *Kdm1a/Lsd1* is considered to be a gene involved in transcriptional repression^19^,^20^. Consistent with this notion, KDM1A/LSD1 recruitment to the *KISS1* and *TAC3* promoters increased markedly after GATAD1 overexpression (Fig. 7l,m), without changing at the *EED* or *EAP1* promoters (Fig. 7n,o). The increased KDM1A recruitment to the *KISS1* and *TAC3* promoters coincided with loss of H3K4me2 (Fig. 7p), without changes in H3K4me3 abundance (Fig. 7q).

**Figure 8:**
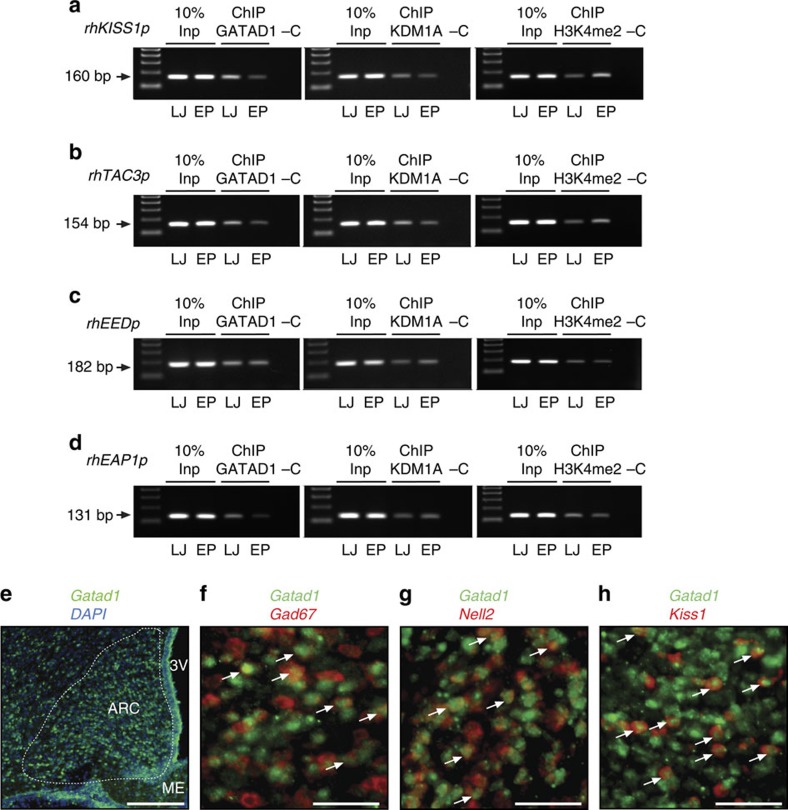
GATAD1 and KDM1A association to the *rhKISS1* and *rhTAC3* promoters. (**a**) Decreased association of GATAD1 and KDM1A to, and increased H3K4me2 abundance at the rhesus (rh) *Kiss1* promoter in the MBH of female monkeys at the juvenile–pubertal transition. (**b**) Similar changes occur at the *rhTAC3* promoter. (**c**) Absence of changes in GATAD1, KDM1A and H3K4me2 at the *rhEED* promoter. (**d**) Reduction of GATAD1 association to the *rhEAP1* promoter in the absence of changes in KDM1A and H3K4me2 abundance. (**e**) Low-magnification image showing the presence of *Gatad1* mRNA transcripts in the ARC of a 28-day-old late juvenile female rat as assessed by fluorescent *in situ* hybridization (FISH) using a *Gatad1* cRNA labelled with FITC. Bar, 200 μm. (**f**) Detection of *Gatad1* mRNA transcripts in a subpopulation of GABAergic neurons (examples denoted by arrows) identified by their content of *GAD67* mRNA (detected using a digoxigenin-labelled *Gad67* cRNA probe). (**g**) *Gatad1* mRNA is also detected in a subpopulation of glutamatergic neurons (arrows) identified by the presence of *Nell2* mRNA (detected with a digoxigenin-labelled *Nell2* cRNA probe). (**h**) Most ARC kisspeptin neurons identified by the presence of *Kiss1* mRNA contain *Gatad1* mRNA transcripts (arrows). Bars in **f**–**h**, 50 μm.

To determine whether the changes in GATAD1 association to the *Kiss1* and *Tac3* promoters observed in cultured cells also occur *in vivo* during the normal initiation of primate puberty, we performed ChIP assays using chromatin derived from the hypothalamus of female rhesus macaques at the LJ and EP stages of pubertal development. The results showed that—consistent with the ChIP results in 293T cells (Fig. 7)—the association of GATAD1 and KDM1A to the *KISS1* and *TAC3* promoters also decreases *in vivo* (Fig. 8a,b), at the time when hypothalamic *GATAD1* mRNA abundance declines (Fig. 3), and coinciding with an increase in the content of the activating histone mark H3K4me2 (Fig. 8a,b). We observed no change in GATAD1, KDM1A or H3K4me2 association to the *EED* promoter (Fig. 8c). Although there was some loss of GATAD1 from the *EAP1* promoter at EP, this change was not accompanied by changes in either KDM1A or H3K3me2 association (Fig. 8d). These results suggest that GATAD1 delays puberty by repressing transcription of *KISS1* and *TAC3* in the ARC, both directly and by reducing the levels of the activating histone mark H3K4me2 at each promoter, via recruitment of KDM1A/LSD1.

To determine whether *Gatad1* transcripts are present in *Kiss1*-containing neurons of the ARC we utilized a double fluorescent *in situ* hybridization technique. We used late juvenile female rats for this study, because we had no available tissue from non-human primates. In addition to kisspeptin neurons, we examined two hypothalamic populations of neurons involved in the control of puberty, GABAergic and glutamatergic neurons. GABAergic neurons contribute to the inhibitory control of GnRH secretion and were visualized by detecting *GAD67* mRNA. Glutamatergic neurons are involved in the excitatory control of GnRH and were visualized by detecting *Nell2* mRNA^21^. As predicted by its transcriptional regulatory functions, *Gatad1* mRNA is expressed in cells throughout the MBH, prominently in the ARC (Fig. 8e). Importantly, *Gatad1* transcripts are present in all three neuronal populations studied (Fig. 8f–h), indicating that the transcriptional regulatory activity of GATAD1 on different genes is controlled by cell-specific factors.

### *GATAD1* and *ZNF573* are connected to menarche genes

To determine whether *GATAD1* and/or *ZNF573* are functionally connected to genes implicated in the timing of human puberty, we used the GeneMANIA literature interaction database^22^ to analyse the known relationships that exist between *ZNF573* and *GATAD1*, and more than 120 genes associated to the age of menarche^11^. When queried, GeneMANIA searches a host of databases describing known gene interactions such as co-expression, shared protein domains, genetic interactions, predicted interactions and shared biochemical and genetic pathways. We observed that this menarche-related gene cohort^11^ is not only densely interconnected, but also connected to *GATAD1* and *ZNF573* (Supplementary Fig. 8a). *GATAD1* and *ZNF573* are significantly (*P*=0.007 and *P*=0.0; permutation test, effective *P* value based on 1,000 replicates) more connected (Supplementary Fig. 8b) to their first neighbours within the overall menarche-related gene network than to 1,000 randomly networks of the same size containing randomly selected gene sets unrelated to menarche, extracted from the GeneMANIA database (Supplementary Fig. 8c). Representation of these connectivity distributions demonstrated that the degree of connectivity of both *GATAD1* and *ZNF573* to menarche-related genes (Supplementary Fig. 8d,e, single red line) was greater than the connectivity observed in random gene set-derived sub-networks (29 vs13.47 and 76 vs14.7, respectively; Supplementary Fig. 8d,e, black lines). It thus appears that *GATAD1* and *ZNF573* are functionally connected to a large cohort of genes that associate with an earlier age of menarche in girls.

## Discussion

The present study provides evidence that members of the ZNF superfamily of transcriptional repressors represent an important component of the neurobiological brake that holds the GnRH pulse generator in check during juvenile development in highly evolved primates, and that, therefore, prevents the premature awakening of the pubertal process in these species. We show that expression of *GATAD1* and *ZNF573*, along with that of five other ZNFs, decreases in the MBH of agonadal male rhesus monkeys during the JUV–PUB transition when GnRH pulse generator activity increases. Similar peripubertal changes in *GATAD1* and *ZNF573* expression were also observed in ovary-intact female monkeys. Moreover, when *GATAD1* or *ZNF573* were overexpressed in the ARC of immature female rats, puberty was delayed and subsequent oestrous cyclicity was disrupted.

Our results show that four of the eight ZNFs displaying decreased expression in the male monkey hypothalamus at puberty (*ZNF573*, *ZNF582*, *ZNF587* and *ZNF791*) belong to the large family of *KRAB–KZNF* genes. These four ‘puberty-related' KRAB–KZNFs are members of the large cluster of KZNFs located in the q13 region of chromosome 19. It is unlikely, however, that all KRAB–KZNFs contribute to silencing genes that at the termination of juvenile development reactivate GnRH pulse generation, as evidenced by the finding that expression of *ZNF483*, a KRAB–KZNF located on chromosome 9, did not decrease during the JUV–PUB transition. Conversely, ZNFs lacking a KRAB domain and located in chromosomes other than chromosome 19 may work in concert with chromosome 19 KRAB–KZNFs to maintain puberty in check. In keeping with this idea, hypothalamic expression of *GATAD1* and *ZNF462*, two ZNFs encoding proteins lacking a KRAB domain, decreased at puberty.

Our results also show that primate-specific ZNFs do not contribute to regulating the timing of puberty in primates. Instead, puberty appears to be regulated by ZNFs, such as *GATAD1* and *ZNF573*, less affected by lineage-specific diversification and expressed across mammalian species, including primates. The term ‘primate-specific' refers to a group of *KRAB–ZNF* and *ZNF* genes that, resulting from lineage-specific evolutionary expansion and diversification, is unique to primates^23^,^24^. The two largest clusters of primate-specific genes reside in the centromeric regions of chromosome 19 (p12–13.1) and chromosome 7 (7p11.2–q11.21). Although we did not detect changes in *GATAD1* expression in the rat hypothalamus at puberty, GATAD1 was effective in delaying puberty in this species suggesting that ZNFs may be able to substitute for other ZNFs in the regulation of major biological processes such as puberty. This idea is supported by the finding of several ZNFs displaying reduced abundance in the monkey hypothalamus at puberty, and is consistent with the concept that the large number of ZNFs present in the mammalian genome is important for the ‘fine tuning of transcriptional regulation within regulatory networks'^25^.

GATAD1 is a particularly intriguing ZNF not only because it has been implicated in determining adult height^14^,^15^, but also because of its contribution to the epigenetic regulation of gene expression^16^. GATAD1 is considered to be a chromatin ‘reader' because it associates to promoter regions containing H3K4me3 to form a repressive complex^16^. H3K4me3 is a *bona fide* histone modification associated with active gene transcription^26^. For the foregoing reasons, we conducted a more detailed analysis of the potential mechanisms by which GATAD1 might induce transcriptional repression in the ARC using a reverse translation approach with the intact female rat serving as our *in vivo* model. This approach revealed that GATAD1 overexpression disrupts the normal changes in gene network connectivity that occur in the ARC as puberty approaches, preventing a juvenile-to-peripuberal organization geared for transcriptional activation and maintaining one less ‘mature' that favours transcriptional silencing. GATAD1-imposed transcriptional silencing appears to be exerted at two levels. The first involves direct transcriptional repression of *Kiss1* and *Tac3*, two genes expressed in the ARC and considered to be central to the pubertal increase in GnRH pulsatility. The second level of transcriptional silencing leads to a switch in the chromatin landscape from a configuration-favouring gene activation to another that promotes gene silencing. The presence of GATAD1 transcripts in kisspeptin neurons of the rat ARC provides morphological support to the concept that GATAD1 is a physiological regulator of the pubertal process.

Expression of both *KISS1* and *TAC3* increases in the monkey hypothalamus, as *GATAD1* expression decreases during the JUV–PUB transition. That these events may be causally related is suggested by the results of our promoter assays showing that GATAD1 inhibits *KISS1* and *TAC3* promoter activity. This repression occurs in the absence of exogenously added co-repressors. Surprisingly, GATAD1 did not affect the promoter activity of other genes tested. This is perhaps due to the need of co-repressors or to a potential effect of GATAD1 on the body of these genes to recruit SETDB1, a methyltransferase that catalyses the synthesis of the repressive histone mark H3K9me3 (ref. 27).

Our gene network analysis predicted the contribution of several epigenetic domains to the regulation of puberty and the repression of this process by GATAD1. We pursued studies on one of these domains, because of its direct relevance to the epigenetic regulation of gene expression. According to our computational analysis, *Kdm1a* loses connectivity in the ARC during normal prepubertal development of the female rat and GATAD1 overexpression prevents this change. KDM1A (also known as LSD1), the lysine demethylase encoded by *Kdm1a*, specifically demethylates H3K4me2 at gene promoters^19^, and, therefore, is linked to gene repression^19^,^20^. Our *in vitro* finding that KDM1A recruitment is increased by GATAD1 overexpression while association of H3K4me2 to the *KISS1* and *TAC3* promoters is dramatically reduced, is consistent with the interpretation that GATAD1 reduces *KISS1* and *TAC3* gene activity in part by facilitating loss of H3K4me2 from these promoters via gain of KDM1A. The decrease in GATAD1 and KDM1A association to the *KISS1* and *TAC3* promoters and the concomitant increase in H2K4me2 abundance observed in the monkey MBH at the time of puberty, strongly support the *in vitro* results and suggest that these epigenetic changes are events normally associated with the time of primate puberty. Interestingly, *GATAD1* and *KDM1A* appear to be interconnected in a broader context as sequence variation in genomic loci harbouring these genes has been shown to affect human height^14^,^15^.

Altogether, these results are consistent with the emerging concept that transcriptional repression of genes involved in the initiation of puberty resides at the heart of the molecular mechanism by which the hypothalamus holds the GnRH pulse generator in check and therefore prevents the premature activation of the pubertal process^4^. The polycomb (PcG) complex, known to play a major role in imposing gene silencing at various developmental stages^28^, was shown by one of our laboratories to prevent the premature initiation of puberty in the rat by repressing the transcriptional activity of *Kiss1* in KNDy neurons^4^. The ability of GATAD1 to promote H3K4me2 demethylation at the *KISS1* and *TAC3* promoters suggests that ZNFs, instead of directly interacting with PcG proteins, may function to counteract the antagonistic effect of the Trithorax complex on PcG-mediated gene silencing. Mono, di and trimethylation of H3 at lysine 4 at target promoters are histone modifications associated with active transcription and catalysed by specific members of the Trithorax complex^29^,^30^.

Of the ZNFs we studied, only expression of GATAD1 exhibited an inverse developmental relationship with the centrally driven ‘on-off-on' pattern of pulsatile GnRH release observed in highly evolved primates during the infant–juvenile and JUV–PUB transitions^1^. This finding implies that the ZNF components of the molecular switch that we posit, controls the pubertal reactivation of GnRH pulsatility, may not be identical to those comprising the switch that represses the GnRH pulse generator during the infant–juvenile transition. Unexpectedly, the decline in GnRH pulsatility during the INF–JUV transition was not accompanied by a decrease in either *KISS1* or *TAC3* mRNA levels. This is surprising in the view of an earlier study showing a greater number of ARC kisspeptin neurons in agonadal infantile male monkeys than in juveniles^31^. Also, hypogonadotropism was reported in an infant boy with a loss-of-function mutation of KISS1R/GPR54 (ref. 32). Clearly additional studies are required to unravel the regulation of GnRH secretion during primate infancy.

By identifying a cohort of ZNFs with decreasing hypothalamic expression during the JUV–PUB transition in rhesus monkeys, and mechanistically characterizing the mode of action of one of them in a rodent model, our results enable us to advance the notion that ZNFs are transcriptional regulators involved in the process by which epigenetic repression contributes to regulating the timing of primate puberty. We further propose that ZNFs may provide a layer of control linked to that provided by *EAP1* and *MKRN3*, two ZNF and RING finger-containing proteins earlier shown to share puberty-regulatory functions in rodents and primates^33^,^34^. Whether a similar ZNF-mediated mechanism contributes to restraining puberty in rodents is not known, but the fact that rat puberty is delayed by human ZNFs suggests this to be the case.

## Methods

### Animals

*Monkeys*. For the study of the INF–JUV transition, 10 male rhesus monkeys bred by the Primate Core of the Center for Research in Reproductive Physiology, University of Pittsburgh were used. Ten juvenile male rhesus monkeys were also obtained from Three Springs Scientific (Perkasie, PA, USA) and maintained at the Center's Primate Core for the study of the JUV–PUB transition. The animals were housed under a controlled photoperiod (lights on, 0700–1900, h), in accordance with the National Institutes of Health (NIH) Guidelines for Care and Use of Laboratory Animals. The University of Pittsburgh Institutional Animal Care and Use Committee approved the experimental procedures. In addition, we used female rhesus monkeys (*Macaca mulatta*) obtained through the Oregon National Primate Research Center (ONPRC) Tissue Distribution Program. As in the case of male monkeys, these animals were classified into different stages of development. This classification was based on the age of the animals and the pubertal stages reported by Watanabe and Terasawa^35^.

*Rats*. We used Sprague Dawley female rats at different phase of postnatal development, including postnatal day (PND)14, EJ period (PND21), LJ period (PND28), peripubertal period (PND29–35) and early adulthood (PND35–50). The use of rats was approved by the ONPRC Animal Care and Use Committee in accordance with the NIH guidelines for the use of animals in research. The animals were obtained from Charles River Laboratories international, Inc. (Wilmington, MA). They were housed in a room with controlled photoperiod (14/10 h light/dark cycle) and temperature (23–25 °C), and were allowed *ad libitum* access to tap water and pelleted rat chow.

### Procedures involving monkeys

*Surgical procedures*. All animals for the study of the INF–JUV transition were bilaterally castrated between 2–6 days after birth using sterile technique under ketamine hydrochloride anaesthesia (40 mg kg^−1^ intramuscularly (i.m.); Ketaject, Phoenix Scientific, Inc., St Joseph, MO, USA) as reported previously^36^. In addition, local anaesthesia at the incision sites was achieved by the application of bupivacaine HCl (Marcaine, 0.5%; Butler Schein Animal Health, Elizabethtown, PA, USA). Immediately before surgery, animals received an i.m. injection of penicillin (Pen-G, 40,000 U kg^−1^; Phoenix Scientific, Inc.) Post-surgically, the infants received an i.m. injection of 2.5 mg flunixin meglumine (Banamine, Butler Schein Animal Health, Elizabethtown, PA, USA) for analgesia. The infants were housed with their mothers in individual cages until they were killed between 1–3 (INF) and 6–11 (JUV) months of age to collect the hypothalamus (see below).

All animals for the study of the JUV–PUB transition were bilaterally castrated as reported previously^37^ under isoflurane anaesthesia (1–2% in oxygen; Abbott Animal House, North Chicago, IL, USA) using sterile technique between 16–18 months of age, that is, 18–20 months before the initiation of puberty would be anticipated had the animals remained intact^38^. Animals were treated daily for 4 days starting on the morning before castration with an i.m. injection of Pen-G (40,000 U kg^−1^). Post-operative analgesia was achieved with twice daily i.m. injection of ketoprofen (Ketofen, 2 mg kg^−1^; Apothecon, Princeton, NJ, USA) for 4 days. Four animals were killed when they were around 19 months of age (JUV), four were killed when they were between 24–30 months of age (Early Pubertal, EP) and two were killed around 31 months of age (Late Pubertal, LP) to collect the hypothalamus. To collect the hypothalamus at the time of killing, animals from both studies were first sedated with an i.m. injection of ketamine (20 mg kg^−1^) followed by deep anaesthesia with intravenous sodium pentobarbital (Nembutal sodium solution, ∼25 mg kg^−1^; Abbott Laboratories, North Chicago, IL, USA) and placed in a stereotaxic head holder. Animals in the INF–JUV study were killed with a lethal intravenous dose of sodium pentobarbital (40 mg kg^−1^). Animals in the JUV–PUB study were killed with a mixture of pentobarbital and phenytoin (Beuthanasia-D, 1 ml kg^−1^; Butler Schein Animal Health, Elizabethtown, PA, USA).

*Blood sampling*. Weekly blood samples (1–2 ml) were collected via femoral venipuncture. For the first 6 months of the INF–JUV study, the mother was sedated with ketamine (20 mg kg^−1^, i.m) to remove the infant for sampling. Subsequently, the mother and infant were both sedated with ketamine to venipuncture the infant. For the JUV–PUB study, all animals were sedated with ketamine (20 mg kg^−1^) for venipuncture. Plasma was separated and stored at −20 °C.

*Tissue dissection*. The brain was removed from the cranium and the hypothalamus isolated as a single block comprised of preoptic area (POA) and MBH. The hypothalamus was then bi-sectioned in the sagittal plane. One half-hypothalamus was further cut along the caudal boundary of the optic chiasm to deliver the POA and MBH. The tissue was rapidly frozen by immersion in liquid nitrogen and stored at −80 °C. A piece of cortical tissue (CTX, negative control) was also dissected, rapidly frozen and stored at −80 °C.

### Procedures involving rats

*Tissue collection*. To measure changes in gene expression that occur in the hypothalamus during juvenile development, the MBH of female rats was dissected by a rostral cut along the posterior border of the optic chiasm, a caudal cut immediately in front of the mammillary bodies, and two lateral cuts half-way between the medial eminence and the hypothalamic sulci. The thickness of the tissue fragment was about 2 mm. On dissection, the tissues were immediately frozen on dry ice and stored at −85 °C until RNA extraction.

*Stereotaxic delivery of lentiviral particles*. To deliver LV particles carrying either a *GATAD1* or a *ZNF573* transgene to the ARC we used 21-day-old EJ female rats, using a procedure previously reported^33^,^39^. The animals were anaesthetized with a cocktail of ketamine (25 mg), xylazine (5 mg) and acepromazine (1 mg), 0.15 ml per 100 g body weight (intraperitoneally), and positioned on a stereotaxic instrument (David Kopf Instruments, Tujunga, CA) with the incisor bar positioned at +5 mm. A total volume of 1 μl of LV-GFP, LV-GATAD1 or LV-ZNF573 was injected bilaterally into the ARC at the rate of 250 nl min^−1^, using a 10 μl Hamilton microsyringe connected to a Stoelting Stereotaxic microinjector (Stoelting, Wood Dale, IL). The coordinates used were: 0.3 mm lateral from midline, 0.2 mm anterior from bregma and 9.6 mm vertical from the surface of the brain, as reported^33^. The surgical procedure lasted about 15 min. Following surgery, the animals were placed in a clean cage on a heating pad until returned to their cages. They were given an analgesic (Carprofen, 5 mg kg^−1^) and an antibiotic (Baytril 10 mg kg^−1^) for 3 days. At the time of killing, the location of the injections was determined by immunohistochemical detection of enhanced GFP (eGFP), as described below.

*Evaluation of sexual maturation and oestrous cyclicity*. To determine the changes in hypothalamic gene expression that occur during the INF–JUV period of sexual development, rats were euthanized at PND14, PND21 and PND28. According to criteria previously established^40^,^41^, PND21 animals are considered to be in the EJ phase of prepubertal development. At this time, the vagina is not yet patent and the uterine weight is 60 mg or less, with no accumulation of intrauterine fluid. At PND28, the rats are in the LJ phase of prepubertal development; their vagina is closed and there are no signs of intrauterine fluid accumulation. Animals in this phase exhibit a diurnal change in pulsatile plasma LH levels, with the LH pulses becoming more pronounced in the afternoons^42^. Like in humans and monkeys, this is the first hormonal manifestation of the increase in central drive that initiates puberty^43^. To determine the effect of intrahypothalamic injections of ZNFs on the onset of puberty and subsequent oestrous cyclicity the animals were inspected daily for vaginal opening starting 5 days after the injections. Once vaginal opening occurred, vaginal lavages were performed daily to identify the occurrence of the first oestrus, which in rodents is manifested by a predominance of cornified cells. Although ovulation normally occurs on the day of oestrous, detection of cornified cells does not indicate that ovulation has occurred, unless vaginal cornification is followed by the appearance of a predominance of leukocytes. The presence of these cells defines the diestrous phase of the oestrous cycle, and indicates that a functional corpus luteum was formed after ovulation. For these reasons, the age at first ovulation was considered to have occurred only when the cornified cells were followed by at least 2 days of lavages containing mostly leukocytes.

### RNA extraction

Total RNA was extracted from the MBH, POA and CTX of male and female rhesus monkeys using the RNeasy maxi kit (Qiagen, Valencia, CA) or from rat MBH using the RNeasy mini kit (Qiagen) following the manufacturer's instructions. To remove DNA contamination, RNA samples were treated by on-column DNAse digestion with the Qiagen RNase-free DNase set according to the manufacturer's protocol. RNA concentrations were determined by spectrophotometric trace (Nanodrop, ThermoScientific, Wilmington, DE).

### Affymetrix arrays

Total RNA from rhesus monkeys was concentrated with the RNA Clean-up Kit-25 (Zymo Research Corporation, Irvine, CA) following the manufacturer's instruction to reach a minimum concentration of 500 ng μl^−1^. RNA integrity was assessed on an Agilent 2100 Bioanalyzer (Agilent Technologies, Inc., Santa Clara, CA) and found acceptable and of equal quality. Samples were amplified, labelled and hybridized to GeneChip Rhesus Macaque Genome Arrays (Affymetrix, Santa Clara, CA). Microarray assays were performed by the Affymetrix Microarray Core of the OHSU Gene Microarray Shared Resource.

### Open array real-time PCR

1,000 ng of total RNA from rat MBH was reverse transcribed (RT) using the Omni RT Kit (Qiagen, Valencia, CA) in the presence of random hexamer primers (Invitrogen, Carlsbad, CA), as recommended by the manufacturer. The resulting complementary DNA (cDNA) was diluted four times with H_2_O and mixed with 2 × TaqMan OpenArray Real-Time PCR Master Mix (Life Technologies, Grand Island, NY) at a ratio of 3.8:1.2 (PCR mix:cDNA). The mix was loaded into custom-made (12 × 224 probes) OpenArray plates (for target genes see Supplementary Data 5; for probe numbers and lengths of amplicon see Supplementary Data 9) using the QuantStudio OpenArray AccuFill platform and the PCR reactions were performed in a QuantStudio 12 K Flex Real-Time PCR System (Applied Biosystems, Foster City, CA). Experiments were performed two times.

### Real-time PCR

400 ng of total RNA were RT as described above. All real-time PCR reactions were performed using a Quanttudio 12 K Real-Time PCR system; threshold cycles (CTs) were detected by QuantStudio 12 K Flex software. Relative standard curves were constructed from serial dilutions of one reference sample cDNA (RT of 500 ng total RNA from one MBH sample serially diluted from 1/2 to 1/500). The CTs from each sample was referred to the relative curve to estimate the mRNA content/sample and the values obtained were normalized for procedural losses using *GAPDH* mRNA (for monkey tissues) or peptidylprolyl isomerase A mRNA (for rat tissues) as the normalizing unit. To determine the relative abundance of the mRNAs of interest, we used the SYBR GreenER qPCR SuperMix system (Invitrogen, Carlsbad, CA). Primers for amplification (Supplementary Data 9) were designed using the PrimerSelect tool of DNASTAR 11 software (Madison, WI) or the NCBI online Primer-Blast program. PCR reactions were performed in a total volume of 10 μl, each reaction containing 1 μl of diluted cDNA or a reference cDNA sample, 5 μl of SYBR GreenER qPCR SuperMix and 4 μl of primers mix (300 nM of each gene specific primer. The PCR conditions used were 95 °C for 5 min, followed by 40 cycles of 15 s at 95 °C and 60 s at 60 °C. To confirm the formation of a single SYBR Green-labelled PCR amplicon, the PCR reaction was followed by a three-step melting curve analysis consisting of 15 s at 95 °C, 15 s 1 min at 60 °C, ramping up to 95 °C at 0.5 °C s^−1^, detection every 0.5 s and finishing 15 s at 95 °C, as recommended by the manufacturer. Experiments were performed three times.

### Affymetrix array analysis

Initial microarray analysis and quality control were performed by the Affymetrix Microarray Core using GCOS version 1.4.0.036 software (Affymetrix). After this initial analysis, the array CEL files provided were uploaded to the GeneSifter analysis platform (http://www.geospiza.com/Products/AnalysisEdition.shtml) for further differential expression and functional analyses. Expression levels were normalized using GeneSifter's implementation of the Guanine–Cytosine robust multiarray analysis algorithm^44^. After filtering for absent probe calls, gene expression levels were visualized using the GeneSifter interface and compared for differential expression across time points using a one-way analysis of variance (ANOVA) analysis. In lieu of Affymetrix standard probe annotation, we used the annotations provided by Robert Nordgren (http://www.unmc.edu/rhesusgenechip/).

### Heat maps and expression density estimation plots

Gene expression data from microarray experiments were analysed in the R statistical environment (R Development Core Team) version 2.15 (http://www.R-project.org/) after data cleaning and normalization with GeneSifter as described above. Gene expressions were transformed to relative expression levels using the mean expression level of late juvenile samples as the reference. Heat maps of gene expression were generated using the heat map function of the gplots package and the RColorBrewer package, while density estimation of changes in the relative level of expression between developmental stages were plotted using R's density function with default parameters.

### Open array analysis

We used the OpenArray qPCR platform to measure changes in relative expression for 224 genes studied during infantile-juvenile development (PND 14, 21 and 28) and after perturbing the system by overexpression of GATAD1 in the ARC (rats injected on PND21 and euthanized on PND28) (Supplementary Data 5 and 6). Raw data were extracted from the QuantStudio 12 K Flex software and analysed using R. CT values were converted to relative expression levels for further analysis using a standard delta–delta transformation. Probes that resulted in 50% or more of undetermined (UD) or flagged replicates were excluded from the study. Data were then used for gene co-expression and network analysis (see below).

### Gene co-expression network analysis

After initial filtering of the OpenArray expression data as described above, we filtered remaining outliers and missing values from the data. The expression of these removed values was inferred with k-nearest neighbour imputation using the impute R package. Due to the two runs of the OpenArray instrument necessary for data collection, genes that were excluded in one run were excluded from the other run, resulting in 147 genes viable for analysis across the three time points and GATAD1 overexpression experiments (Supplementary Data 6). The remaining expression values were then corrected for batch effects using the ComBat method of Johnson *et al*.^45^ as implemented in the sva R package. Thereafter, we constructed gene co-expression networks for each individual time point and the GATAD1 overexpression data set using a compressive-sensing-based network ensemble methodology developed by our group. We generated all possible one-gene Pearson partial correlation matrices (for example, for *n* genes, *n* matrices are produced, with the *n*th matrix representing the pairwise partial correlations of expression for all genes other than *n* when accounting for the correlation of *n* with both genes in a pair) from the data using the R ppcor package. We then utilized the CLIME algorithm^46^ as implemented in the R package clime to approximate inverse matrices for each partial correlation matrix in the ensemble. Twenty inverse matrices were estimated for each partial correlation matrix for twenty equi-spaced values of the CLIME regularization parameter lambda between 1 and 0.01. We then selected the inverse matrix from the set that resulted in a network with a node degree distribution best approximating a scale-free distribution, filtered the edges in the resultant network based on a cutoff threshold of the 10th percentile of absolute edge weight in the inverse matrix. The lambda range and cutoff value were determined using empirical analysis of *in silico* simulated expression data (unpublished) to minimize false positives and negatives. Co-expression relationships that occur in 95% or more of the resultant networks were then included in the overall network (Supplementary Data 10), under the premise that edges appearing in this fraction of the total occurrences of all edges in the derived network are more likely to represent relatively direct or extremely strong co-expression relationships between the genes rather than spurious correlations or coregulation by other factors. Networks were visualized, analysed and compared using R and Cytoscape 3.1.1 (www.cytoscape.org).

### Functional annotation of gene sets

In addition to analysing the structure of individual networks, the top 20 genes (including ties for the 20th position) losing or gaining network connectivity during prepubertal development were selected for Gene Ontology enrichment analysis using the DAVID tool^47^,^48^; gain and loss were defined as the sum of changes in node degree for a gene between each pair of the networks derived from the three time points assessed. In addition, we repeated this procedure for the top 20 genes losing or gaining connectivity in the network inferred from the GATAD1 overexpression data set as compared with the PND28 network. Overrepresented annotation categories for each set of genes were defined as categories with a *P* value of 0.05 or less as reported by DAVID's modified Fisher Exact Test procedure after Benjamini–Hochberg correction for multiple testing was applied. Genes were mapped to human orthologs due to the superior functional annotation of the human genome, and compared against the human annotation as a background.

### Preparation of lentiviruses for ZNF overexpression

To generate lentiviral particles expressing human GATAD1 or human ZNF573 the coding regions of these genes were purchased from OriGene Technologies (Rockville, MD). Both were provided as plasmids, GATAD1 inserted into the *Eco*RI–*Xho*I sites of pCMV6-AC, and ZNF573 into the *Sgf*l–*Mlu*I sites of pCMV-Entry (RC209422). GATAD1 was cloned into the *Bam*HI–*Sma*I sites of the lentiviral vector LV-Ief via In-Fusion PCR cloning (http://www.clontech.com/US/Products/Cloning_and_Competent_Cells/Cloning_Kits/Cloning_Kits-HD-Liquid) using the primers described in Supplementary Data 9. These primers encompass the juncture between the *GATAD1* cDNA and LV-Ief. The LV-IEF plasmid derives from the plasmid pLVI IRES^49^,^50^ in which the IRES element connecting the gene of interest to eGFP was replaced with the promoter of human elongation factor to drive eGFP expression independent of the inserted gene. pLVI-IRES is a third generation plasmid, in which the promoter sequences of the 5′-LTR were replaced by the cytomegalovirus (CMV) promoter resulting in the formation of a heterologous U3 promoter^51^,^52^. *ZNF573* was excised from pCMV-Entry by first linearizing the plasmid with *Fse*I and then blunting the *Fse*I site with T4 DNA polymerase followed by *Bam*HI digestion. The resulting fragment was then cloned into the *Bam*HI–*Sma*I sites of LV-Ief. The coding region of h*GATAD1* is tagged with the human influenza HA and ZNF573 is tagged with both MYC and a FLAG epitope for easy detection by western immunoblotting. To enhance transgene expression^53^, we inserted the rat insulin II intron A sequence^54^ between the CMV promoter and the *GATAD1/ZNF573* cDNAs. This heterologous intron has been previously used in transgenic mice^53^,^55^.

Infective lentiviral particles were produced as previously described^33^,^49^. In brief, we used the calcium phosphate method to cotransfect 293T cells with four plasmids; the LV vector, pLP1, pLP2 and pLPv (Invitrogen). Plasmid pLP1 expresses *gag* and *pol*; pLP2 expresses *Rev*; and pLPv expresses the VSVG envelope protein. The viral concentration was increased by ultracentrifugation, and the titre of the viral stock was determined by infection of naive 293T cells. Following infection with serial dilutions of the viral stock, the percentage of GFP-positive (fluorescent) cells was determined by flow cytometry. The titre was expressed as transducing units per millilitre (TU ml^−1^).

### Functional promoter assays

To determine whether ZNF proteins alter the transcriptional activity of putative target genes (*KISS1*, *TAC3*, *Ttf1*, *Eed*, *EAP1*, *PEnk* and *Viaat*) we transfected Neuro2A cells (N2A, ATCC, Manassas, VA) with luciferase reporter constructs^56^ containing the 5′ flanking region of these genes, in addition to the LV-GATAD1 or LV-ZNF573 constructs mentioned above. The cells (ATCC, Manassas, VA) were cultured in a humidified atmosphere containing 5% CO_2_ and 37 °C. They were maintained in DMEM containing high glucose (4.5 g l^−1^; Sigma), supplemented with 10% fetal bovine serum (Invitrogen), Glutamine (Sigma), 100 U ml^−1^ penicillin, and 100 μg ml^−1^ streptomycin (Invitrogen). For the assays, the cells (400,000 cells per well) were seeded onto 24-well plates in DMEM containing 10% fetal bovine serum. Twenty-four hours later, the reporter constructs (all of them in the luciferase reporter plasmid pGL2) were transiently co-transfected along with LV-GATAD1 or LV-ZNF573 for 5 h using Lipofectamine 2000 (Invitrogen) at a ratio (1 μg DNA:2.5 μl Lipofectamine 2000) in Optimem (Invitrogen). After transfection, the cells were returned to serum containing DMEM medium; 24 h later, they were harvested and assayed for luciferase activity using the Firefly Luciferase Glow Assay Kit (Pierce, Rockford, IL). The assay was performed in opaque 96-well plates and light emission measured in a Spectramax M5 microplate reader (Molecular Devices, Sunnyvale, CA). Transfection efficiency was normalized by co-transfecting the plasmid CMV-Sport-β-gal (Invitrogen) at 10 ng ml^−1^ and determining β-Galactosidase activity using the Tropix Galacto Light Plus (ABI) as reported earlier^33^. Experiments were performed three times.

### Western blots

To verify the ability of the LV-GATAD1 and LV-ZNF573 constructs to produce a mature protein, we transfected 293T cells with the constructs using Lipofectamine 2000 (Invitrogen) and lysed the cells 48 h later with 500 μl of freshly prepared RIPA buffer (25 mM Tris, pH 7.4, 1% Triton X-100, 1% sodium deoxycholate, 0.1% SDS, 150 mM NaCl, 50 mM β-glycerophosphate, 1 mM sodium pyrophosphate, 1 mM sodium orthovanadate, 10 μg ml^−1^ leupeptin and Pepstatin A, 10 μg ml^−1^ aprotinin and 100 μg ml^−1^ PMSF). Protein concentrations were estimated using the Pierce 660 nm Protein Assay (Pierce, Rockford, IL). Samples were boiled for 5 min in sample buffer and then fractionated in an 8–16% precast SDS–PAGE gel (Invitrogen, Carlsbad, CA). After electrophoresis at 130 V for 2 h, the proteins were transferred for 4 h at 4 °C onto a polyvinylidene difluoride membrane (Millipore, Billerica, MA). The membrane was blocked in 5% non-fat milk for 1 h, before adding the appropriate antibodies (Supplementary Table 1). After an overnight incubation at 4 °C with the primary antibody, the membranes were incubated with a species-specific anti IgG-horseradish peroxidase antibody (1 h at room temperature, 1:10,000; Invitrogen). The signal was developed by enhanced chemiluminescence using the western lightning chemiluminescence substrate (Pierce).

### ChIP assay

To assess the recruitment of GATAD1 to target-specific gene promoters, as well as the association of histone marks to these regions, we performed ChIP assays using chromatin extracted from either 293T cells transfected with the HA-tagged LV-ODAG construct, or hypothalamic tissue (MBH) derived from two female rhesus monkeys. One of these animals (2 years and 99 days age) was in the late juvenile phase of postnatal development; the other (3 years and 73 days of age) was in the early puberty phase. The ChIP procedure was described previously by us^4^,^56^,^57^, and was carried out with minimal modifications. Cells were harvested for ChIP 48 h after transfection. The cells were washed once in ice-cold PBS containing a protease inhibitor cocktail (1 mM phenylmethylsulfonylfluoride, 7 μg ml^−1^ aprotinin, 0.7 μg ml^−1^ pepstatin A, 0.5 μg ml^−1^ leupeptin), a phosphatase inhibitor cocktail (1 mM β-glycerophosphate, 1 mM sodium pyrophosphate and 1 mM sodium fluoride), an HDAC inhibitor (20 mM sodium butyrate). Cross-linking was performed by incubating the cell suspension or MBH fragments in 1% formaldehyde for 10 min at room temperature. After two additional washing steps in PBS, the cells and MBH fragments were lysed with 200 μl SDS buffer (0.5% SDS, 50 mM Tris-HCl, 10 mM EDTA) containing protease, phosphatase and HDAC inhibitors and sonicated for 45 s to yield chromatin fragments of ∼500 bp using a Fisher Scientific FB 705 sonicator. Size fragmentation was confirmed by agarose gel electrophoresis. The sonicated chromatin was clarified by centrifugation at 16,200 *g* for 10 min at 4 °C, brought up to 1 ml in Chip Dilution Buffer (16.7 mM Tris-HCl, pH 8.1, 150 mM NaCl, 1.2 mM EDTA, 1.1% Triton X-100 and 0.01% SDS) containing protease, phosphatase and HDAC inhibitors and stored at −80 °C for subsequent IP. For this step, chromatin was pre-cleared with Protein A/G beads (Dynabeads, Invitrogen, Carlsbad, CA) for 1 h at 4 °C. 100 μl aliquots of chromatin were then incubated with 5 μg antibodies. The antibodies used are described in Supplementary Table 1. Antibody–chromatin complexes and 25 μl of protein A or G beads solution (Dynabeads) were incubated at 4 °C overnight with gentle agitation. Immunocomplexes were washed sequentially with 0.5 ml low-salt wash buffer (20 mM Tris-HCl, pH 8.1, 150 mM NaCl, 2 mM EDTA, 1% Triton X-100 and 0.1% SDS), high-salt wash buffer (20 mM Tris-HCl, pH 8.1, 500 mM NaCl, 2 mM EDTA, 1% Triton X-100 and 0.1% SDS), LiCl buffer (10 mM Tris-HCl, pH 8.1, 250 M LiCl, 1% Nonidet P-40, 1% sodium deoxycholate and 1 mM EDTA) and with TE buffer (10 mM Tris-HCl, pH 8.0 and 1 mM EDTA). The immunocomplexes were eluted with 100 μl of 0.1 M NaHCO3 and 1% SDS at 65 °C for 45 min. Cross-linking was reversed by adding 4 μl of 5 M NaCl and incubating at 95 °C for 30 min. DNA was recovered by using the ChIP DNA Clean and Concentrator columns (Zymo Research, Irvine, CA) and stored at −80 °C until subsequent PCR analysis. All chemicals were purchased from Sigma-Aldrich (St Louis, MO, USA).

### PCR detection of ChIP DNA

Genomic regions of interest were amplified by qPCR. Accession numbers of the genes analysed as well as the chromosomal position of the 5′-flanking region amplified, using the position of the transcription start site as the reference point, are shown in Supplementary Data 9. The primer sequences (Eurofins MWG Operon, Huntsville, AL) used to detect the DNA fragment of interest in the immunoprecipitated DNA are also shown in Supplementary Data 9. For semi quantitative detection, PCR reactions were performed using 1 μl of each IP and Input samples and SybrGreen ER (Invitrogen, Carlsbad, CA) in a volume of 10 μl. The thermocycling conditions used were: 95 °C for 5 min, followed by 40 cycles of 15 s at 95 °C and 60 s at 60 °C. Data are expressed as % of IP signal / Input signal. Experiments were performed three times. For qualitative detection, PCR reactions were performed using 1 μl of each IP and Input samples and HotStart Taq polymerase (Qiagen, Valencia, CA) in a volume of 25 μl. The thermocycling conditions used were: 95 °C for 5 min, followed by 35 cycles of 30 s at 95 °C, 60 s at 60 °C and 60 s at 72 °C. 12 μl of PCR product were run in a 1.6% Agarose-TBE gel (uncropped images are supplied as Supplementary Figs 9 and 10).

### Immunohistofluorescence

To verify the location of microinjections targeting GFP-expressing lentiviruses to the ARC of the hypothalamus, we perfusion-fixed the brain of the injected rats 22–25 days after the initial infection with 4% paraformaldehyde-PBS pH 7.4, blocked the portion of the brain containing the hypothalamus, and serially sectioned the blocks at 30 μm intervals. The sections were then incubated overnight at 4 °C with goat polyclonal antibodies against GFP (Abcam, Cambridge, MA; 1:2,000 dilution), and the reaction was developed the next day to a green colour using Alexa 488 donkey antigoat IgG (Invitrogen, 1:500). Fluorescent images were acquired with an AxioImager A2 Zeiss fluorescent microscope.

### Fluorescent *in situ* hybridization

The brains from two late juvenile 28-day-old female rats were fixed by intracardiac perfusion of 4% paraformaldehyde borate buffer, pH 9.5, and were processed for hybridization histochemistry, as previously described^58^,^59^,^60^,^61^. We used the double fluorescence *in situ* hybridization procedure described by Watakabe *et al*.^62^ employing four different cRNA probes. Three of them (*rKiss1*, *rGad67* and *rNell 2*) were labelled with digoxigenin-11-UTP (Dig). The fourth probe (r*Gatad1*) was labelled with fluorescein-12-UTP (FITC). The *Kiss1* cRNA probe was transcribed from a 379 bp rat *Kiss1* cDNA corresponding to nucleotides (nt) 1–379 in rat *Kiss1* mRNA^63^, the *GAD67* cRNA was generated by *in vitro* transcription of a 220 bp cDNA template complementary to nt 303–523 in the coding region of rat *Gad67* mRNA^64^, and the *Nell2* cRNA probe was transcribed from a 333 bp cDNA corresponding to nt 548–880 in rat *Nell2* mRNA^21^. Finally, the r*Gatad1* cRNA probe was transcribed from a cDNA template corresponding to nt 223–570 in the coding region of r*Gatad1* mRNA. The labelling reactions were performed in a 10 μl volume, as reported^65^. Control sections were incubated with sense probes transcribed from the same plasmid, but linearized on the 3′-end to transcribe the coding strand of the cDNA template. The fluorescent *in situ* hybridization procedure used was identical to that previously described by our laboratory^4^.

### Gonadotropin assays

Plasma levels of LH and FSH were determined using homologous RIAs described previously^66^,^67^. For the LH assay, recombinant cynomolgus (rc) LH (AFP6936A) was used as the standard and radioiodinated trace and a polyclonal rabbit antiserum (AFP342994) raised against rcLH as the first antibody. For the FSH assay, rcFSH (AFP6940A) was used as the standard and radioiodinated trace and a polyclonal rabbit antiserum (AFP782594) raised against rcFSH as the first antibody. The mean sensitivity of the LH and FSH assay ranged between 0.15–1.5 ng ml^−1^ and 0.1–0.7 ng ml^−1^, respectively, depending on the volume of plasma used for the determination. Intra- and inter-assay coefficients of variation were less than 6.9 and 15.8% for LH and 9.8 and 15.3% for FSH, respectively. Gonadotropin concentrations below detection were assigned a value equivalent to the minimum detectable concentration.

### Statistics

All statistical analyses were performed using SigmaStat software (Systat Software Inc., San Jose, CA). The differences between several groups were analysed by ANOVA followed by the Student–Newman–Keuls multiple comparison test for unequal replications. The Student's *t*-test was used to compare two groups. When comparing percentages, groups were subjected to arc-sine transformation before statistical analysis to convert them from a binomial to a normal distribution^68^. For an overall analysis of the real-time PCR data, the results were analysed using paired Student's *t*-tests (two groups). Statistical significance of the connectivity that exists between ZNF573 and GATAD1 to menarche-associated genes was assessed relative to the distribution of their connectivity in an ensemble of 1,000 networks based on randomly selected gene set as described^69^. Significance of the overlap detected between the top 20 genes gaining and losing connectivity over the PND14/21/28 co-expression networks and the top 20 genes gaining and losing connectivity between the GATAD1 overexpression and PND28 co-expression networks was assessed by using a hypergeometric test to determine the probability of selecting the same number of overlapping genes in 20 draws from 147 genes without replacement by chance. A *P* value of <0.05 was considered statistically significant. The sample size was selected based on power analyses performed using the s.d. that we normally observe when measuring the parameters examined in this study and an *n*=6 per group. These analyses provide at least 80% (type II error=0.124) power to detect two effect sizes using either ANOVA or two-sided two-sample *t*-test with a significance level of 0.05.

## Additional information

**Accession codes:** The microarray data has been deposited into Gene Expression Omnibus hosted at the National Center for Biotechnical Information with the accession numbers GSE64396 and GSE64347.

**How to cite this article:** Lomniczi, A. *et al*. Epigenetic regulation of puberty via Zinc finger protein-mediated transcriptional repression. *Nat. Commun.* 6:10195 doi: 10.1038/ncomms10195 (2015).

## Supplementary Material

Supplementary Information Supplementary Figures 1-10, Supplementary Table 1 and Supplementary References

Supplementary Data 1 DAVID functional annotation of genes downregulated during juvenile-pubertal transition in the medial basal hypothalamus (MBH) of agonadal male macaques as assessed by DNA microarrays.

Supplementary Data 2 List of ZNF genes used to generate gene expression heatmaps and density plots of mRNA levels (Fig. 2) derived from the interrogation of the MBH from agonadal juvenile-pubertal male macaques via Affymetrix DNA microarrays.

Supplementary Data 3 Changes in hypothalamic gene expression at the time of the juvenile-pubertal transition in agonadal male monkeys as detected by DNA arrays.

Supplementary Data 4 Expression of genes shown to be involved or potentially implicated in the onset of puberty during the infantile-early juvenile transition in the MBH of agonadal male rhesus monkeys, as assessed by DNA arrays.

Supplementary Data 5 Genes included in a custom made TaqMan Openarray plates (Applied Biosystems) used to assess changes in expression of a set of 224 genes involved in diverse cellular functions, including trans-synaptic communication, glia-toneuron communication, cell adhesiveness, transcriptional control and epigenetic regulation.

Supplementary Data 6 Changes in gene expression measured by the OpenArray platform in the MBH of female rats at three phases of prepubertal development (infantile-PND14, early juvenile [EJ] - PND21 and late juvenile [LJ] - PND28), and seven days after targeting GATAD1 to this region in PND21 rats.

Supplementary Data 7 (A) Functional enrichment of genes gaining connectivity in inferred coexpression networks from PND14 to PND28 in the MBH of prepubertal female rats. (B) Functional enrichment of genes losing connectivity from PND14 to PND28 in inferred coexpression networks.

Supplementary Data 8 (A) Functional annotation of the top 20 genes losing connectivity in the MBH of prepubertal female rats seven days after GATAD1 overexpression in comparison to rats at PND 28 injected with a control GFP expressing lentiviral construct. (B) Functional annotation of the top 20 genes gaining connectivity after GATAD1 overexpression.

Supplementary Data 9 Primers used to detect mRNA levels by RT-PCR, DNA enrichment by ChIP-PCR, and to clone human GATAD1.

Supplementary Data 10 Connectivity analysis of genes assayed for expression using the OpenArray platform and employed in inferred co-expression networks at different times of prepubertal development (PND14, 21, and 28) and at PND28, seven days after instituting GATAD1 overexpression.
