# Supplementary material for: Epigenetic regulation of puberty via Zinc finger protein-mediated transcriptional repression
Source: Nat Commun. 2015 Dec 16;6:10195. doi: 10.1038/ncomms10195 (PMC4703871; doi:10.1038/ncomms10195)
Supplement: Supplementary Information — Supplementary Figures 1-10, Supplementary Table 1 and Supplementary References [file ncomms10195-s1.pdf]

**Supplementary Figure 1**

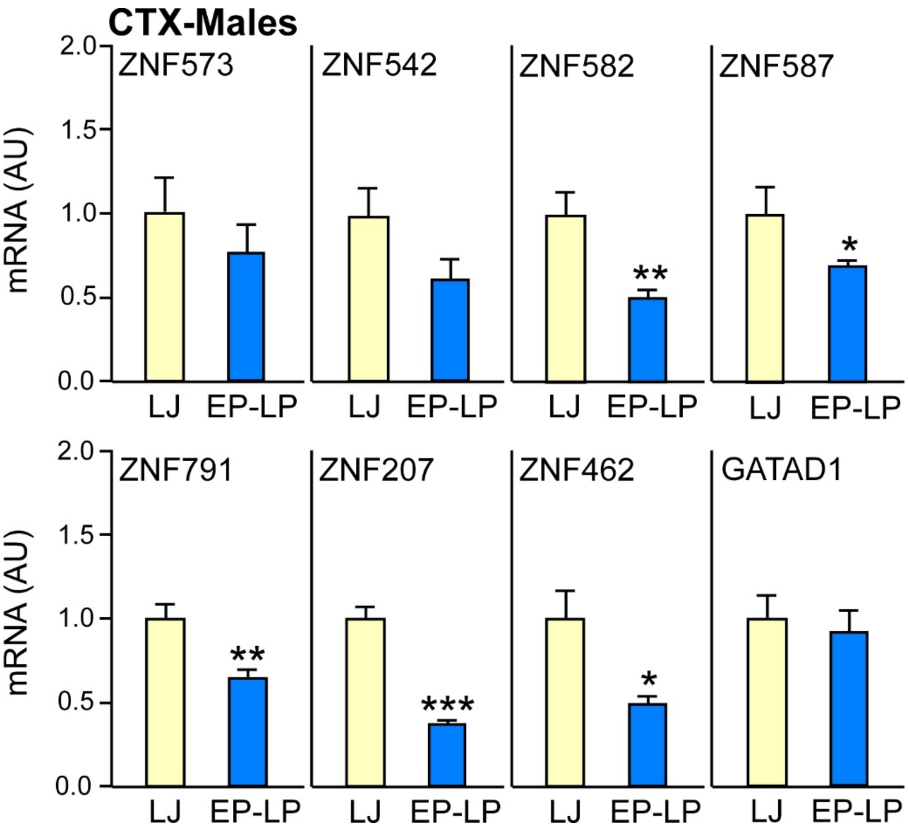


**Supplementary Figure 1: ZNF mRNA levels in the monkey cerebral cortex at the juvenile-pubertal transition.** During this developmental transition low levels of circulating gonadotropins begin to increase reflecting the reactivation of GnRH pulse generation that triggers puberty in intact monkeys (see Fig. 1). All animals were orchidectomized at 16-18 months of age. Because mRNA levels did not change further in LP as compared with EP these two groups were combined before statistical analysis. Vertical bars are Means ± SEM. LJ = late juvenile (n=4-6); EP = early puberty and LP = late puberty (n=5-8) (*p<0.05, **p<0.01 and ***p<0.001 vs. LJ group; Student's t-Test).

**Supplementary Figure 2**

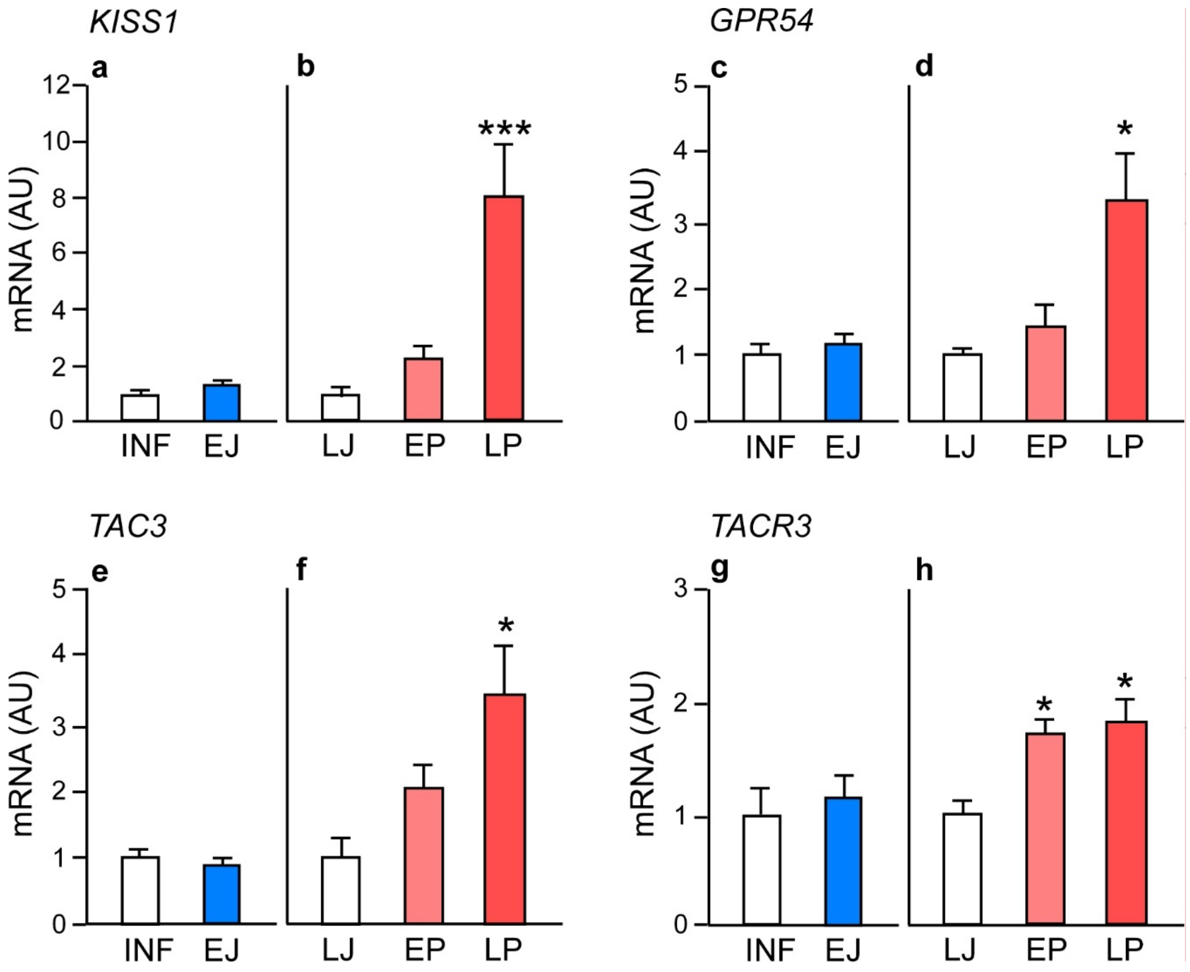


**Supplementary Figure 2: Changes in *KISS1/GPR54* and *TAC3/TACR3* expression in the monkey MBH during postnatal development. a)** *KISS1* mRNA levels do not change during the infantile-juvenile transition, but **b)** increase markedly during the juvenile-pubertal transition. **c)** *GPR54* mRNA levels also remain unaltered during the infantile-juvenile transition, and **d)** increase during the juvenile-pubertal transition. **e)** *TAC3* mRNA levels do not change during the infantile-juvenile transition, but **f)** increase during the juvenile-pubertal transition. **g)** *TACR3* mRNA levels also remain unchanged during the infantile-juvenile transition and **h)** increase at puberty. Vertical bars are means± SEM. INF = infantile phase of development (n=3-5); EJ = early juvenile (n=4-

5); LJ = late juvenile (n=4-6); EP = early puberty (n=4-5); LP = late puberty (n=3-4). In **b)** *** = p<0.001 vs. LJ and EP groups; in **d)** * = p<0.05 vs. LJ and EP groups, in **f,h)** * = p<0.05 vs. LJ group. One Way ANOVA, Student Newman Keuls *post hoc* test).

**Supplementary Figure 3**

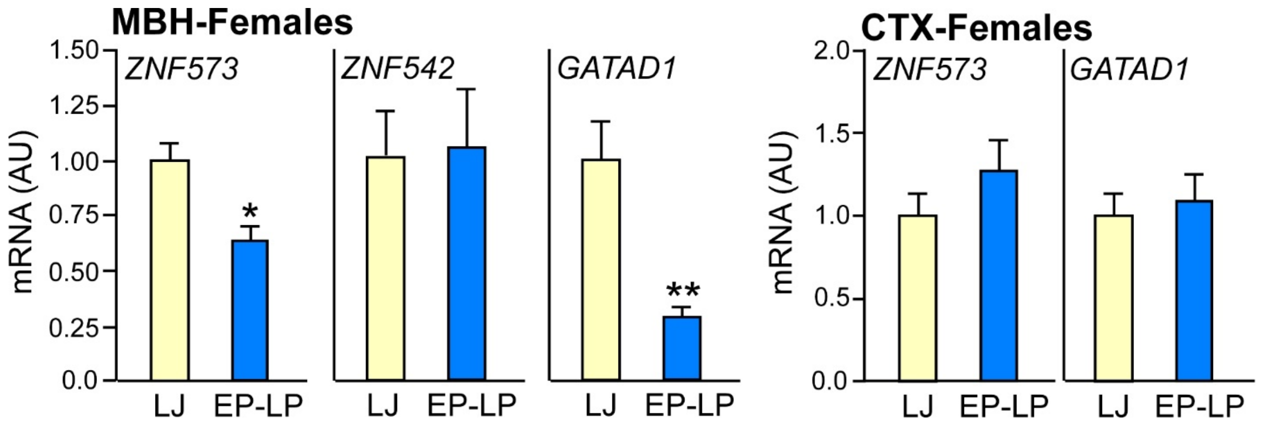


**Supplementary Figure 3: ZNF mRNA levels in the female monkey MBH and CTX at the juvenile-pubertal transition.** At this time low levels of circulating gonadotropins begin to increase towards pubertal values reflecting the pubertal reinitiation of pulsatile GnRH release [1]. EP and LP groups were combined for statistical purposes. ** = p<0.01 vs. LJ group. Vertical bars are Means ± SEM. LJ = late juvenile (n=4-6); EP = early puberty and LP = late puberty (n=5-8) (*p<0.05, **p<0.01 and ***p<0.001 vs. LJ group; Student's t-Test).

**Supplementary Figure 4**

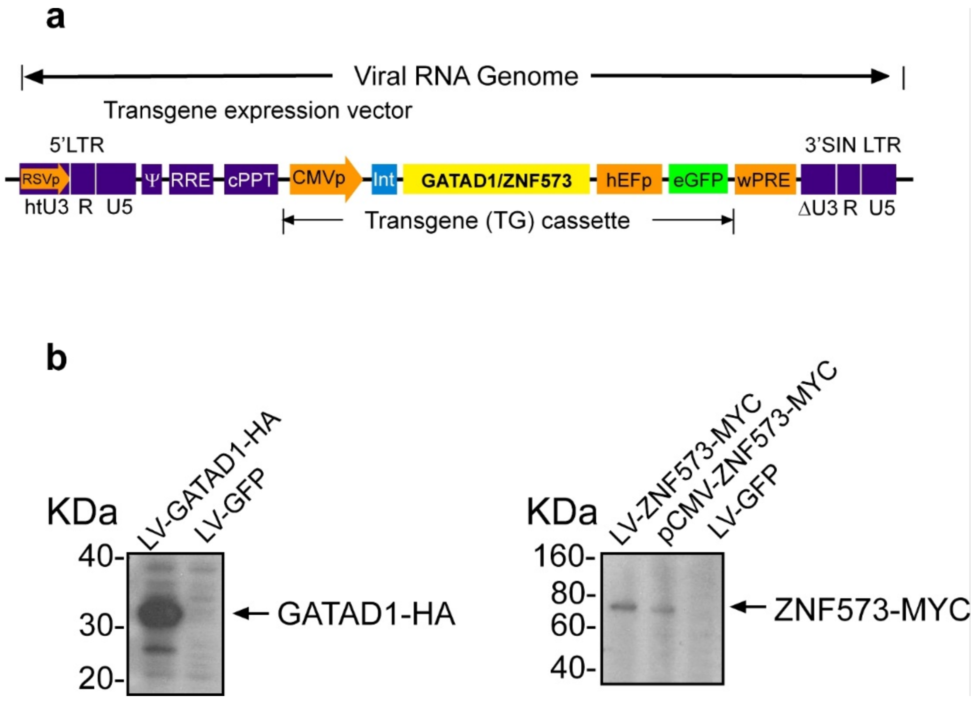


**Supplementary Figure 4: Preparation and testing of a lentivirus construct encoding GATAD1 or ZNF573-tagged proteins.** (**a**) Map of the 3rd generation lentiviral vector used to express human GATAD1 tagged with the HA epitope or human ZNF573 tagged with MYC-DDK epitopes. The 5'LTR contains the heterologous U3 promoter (htU3), the repeat region/transcription initiation site (R), and the polyadenylation region (U5). The R and U5 regions are also present in the 3'LTR, but there is a 400 bp deletion in the 3'LTR U3 region (ΔU3) that results in vector self-inactivation (SIN). The heterologous promoters are either the Rous sarcoma virus promoter (RSVp) or the cytomegalovirus promoter (CMVp); the CMVp also serves as the transgene cassette promoter. The other components include the packaging signal (ψ), the Rev response element binding site (RRE), the central polypurine tract (cPPT), and the woodchuck-hepatitis-virus posttranslational regulatory element (wPRE). The vector contains two transgene cassettes, one composed of HA-tagged human GATAD1 or MYC-DDK tagged human ZNF573

under the control of a CMV promoter, and another consisting of an enhanced green fluorescent protein (eGFP) cDNA under the transcriptional control of the human elongation factor (hEF) promoter. (**b**) Detection of a ~30 kDa band corresponding to GATAD1 and a ~78 kDa band corresponding to ZNF573-MYC-DDK by Western immunoblot of protein extracts derived from 293T cells transfected with the LV-GATAD1-HA or LV-ZNF573-MYC-DDK lentiviral construct. LV-GATAD1-HA: cells transfected with the lentiviral vector encoding human GATAD1 and GFP, LV-ZNF573-MYC-DDK: cells transfected with the lentiviral vector encoding human ZNF573 and GFP; pCMV-ZNF573-MYC-DDK: cells transfected with pCMV-Entry (RC209422) encoding human ZNF573 (Origene); LV-GFP: cells transfected with the empty lentiviral vector carrying only the green fluorescent protein (GFP).

**Supplementary Figure 5**

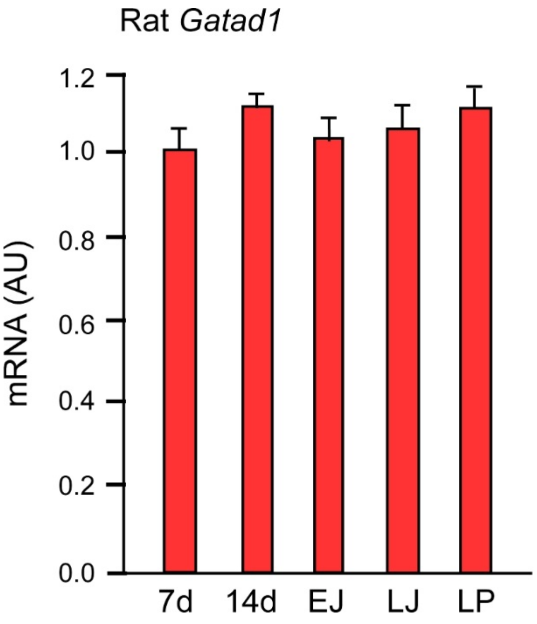


**Supplementary Figure 5: GATAD1 mRNA in rat MBH before puberty.** *GATAD1* mRNA levels do not change significantly in rat MBH during postnatal development. EJ = early juvenile, PND21; LJ = late juvenile, PND28, LP = late proestrous phase of puberty; this is the day when the first preovulatory surge of gonadotropins take place. Bars are means and vertical lines represent SEM. (n = 6-10 per group)

**Supplementary Figure 6**

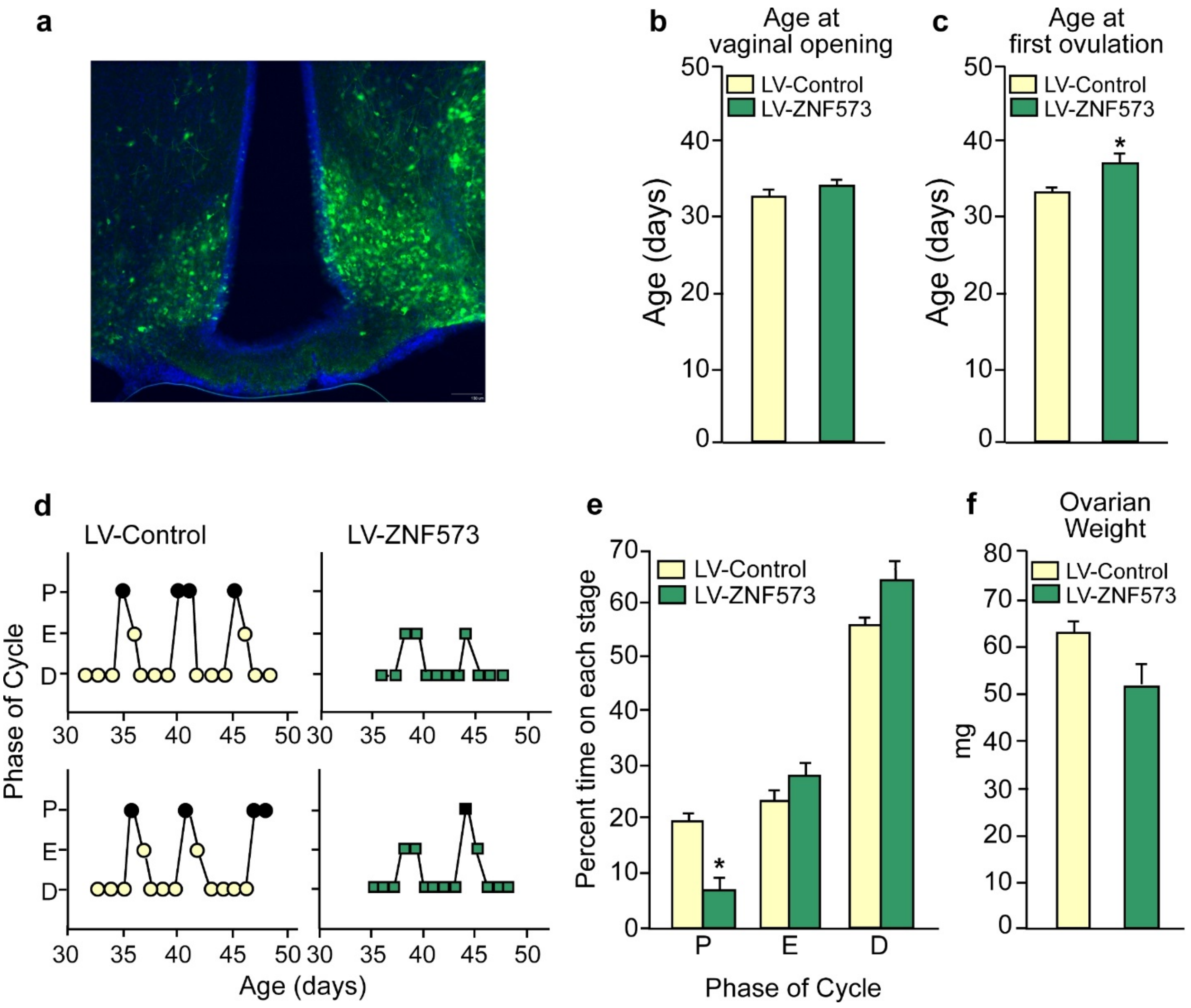


**Supplementary Figure 6: Overexpression of h*ZNF573* in the ARC of immature female rats.** **a,** Immunohistofluorescent localization of GFP-expressing cells in the ARC of a female rat 30 days after receiving a lentivirus construct expressing h*ZNF573* (LV-ZNF573). The construct was stereotaxically delivered to the ARC on PND21. A coronal plane of the MBH is depicted. Bar = 200 µm. Rats injected with an *LV-GFP* construct or rats with misplaced *LV-GATAD1* injections (too high or too lateral) behave similarly, and therefore were combined into a single group (LV-Control) for statistical purposes. **b)** The age at vaginal opening is not significantly delayed in *LV-*

*GATAD1*- injected rats. **c)** The age of first ovulation is delayed in these animals as compared to control rats. (*p<0.05 vs LV-Control group; Student's t-Test)(n=6 per experimental group) **d)** Examples of disrupted estrous cyclicity in *LV-ZNF573* injected rats. **e)** Percent of time spent in different stages of the estrous cycles by rats injected with *LV-ZNF573* in the ARC (*LV-ZNF573*) vs. control animals (LV-Control) (*p<0.05 vs LV-Control group; Student's t-Test) (n=6 per experimental group) **f)** Ovarian weight did not decrease significantly in *LV-ZNF573* injected rats. Vertical bars are Means ± SEM. * = p<0.05 and ** = p<0.01 vs LV-Control group. ME = median eminence; 3V = third ventricle. P = proestrous; E = estrous; D = diestrous.

**Supplementary Figure 7**

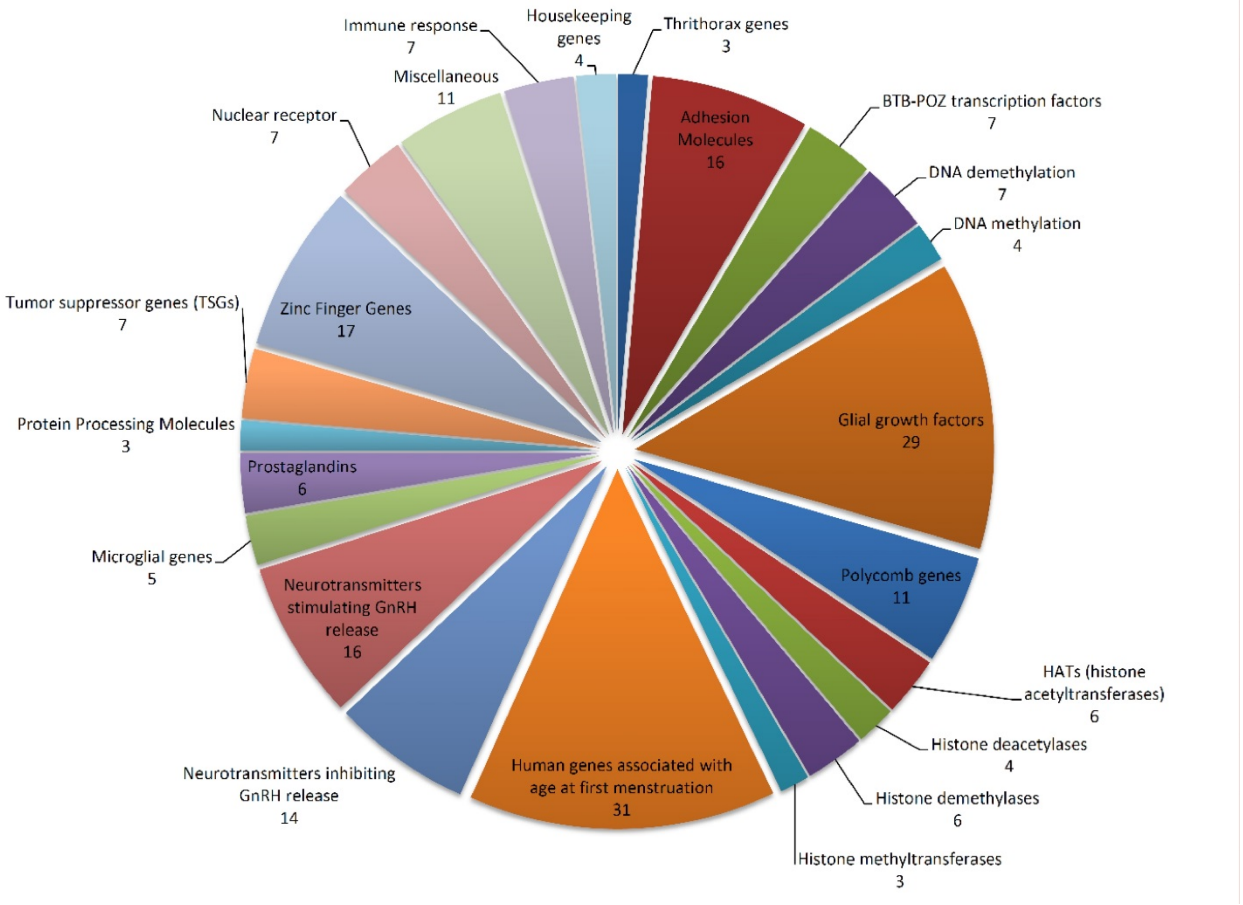


**Supplementary Figure 7: Functional categories of genes selected for analysis by Open Array-RealTime PCR**. A set of 224 genes examined using the Open Array-RealTime PCR system can be distributed into 23 functional categories according to their cellular, molecular or physiological functions. These genes were selected because they have an either established or purported involvement in the neuroendocrine control of pubertal development

**Supplementary Figure 8**

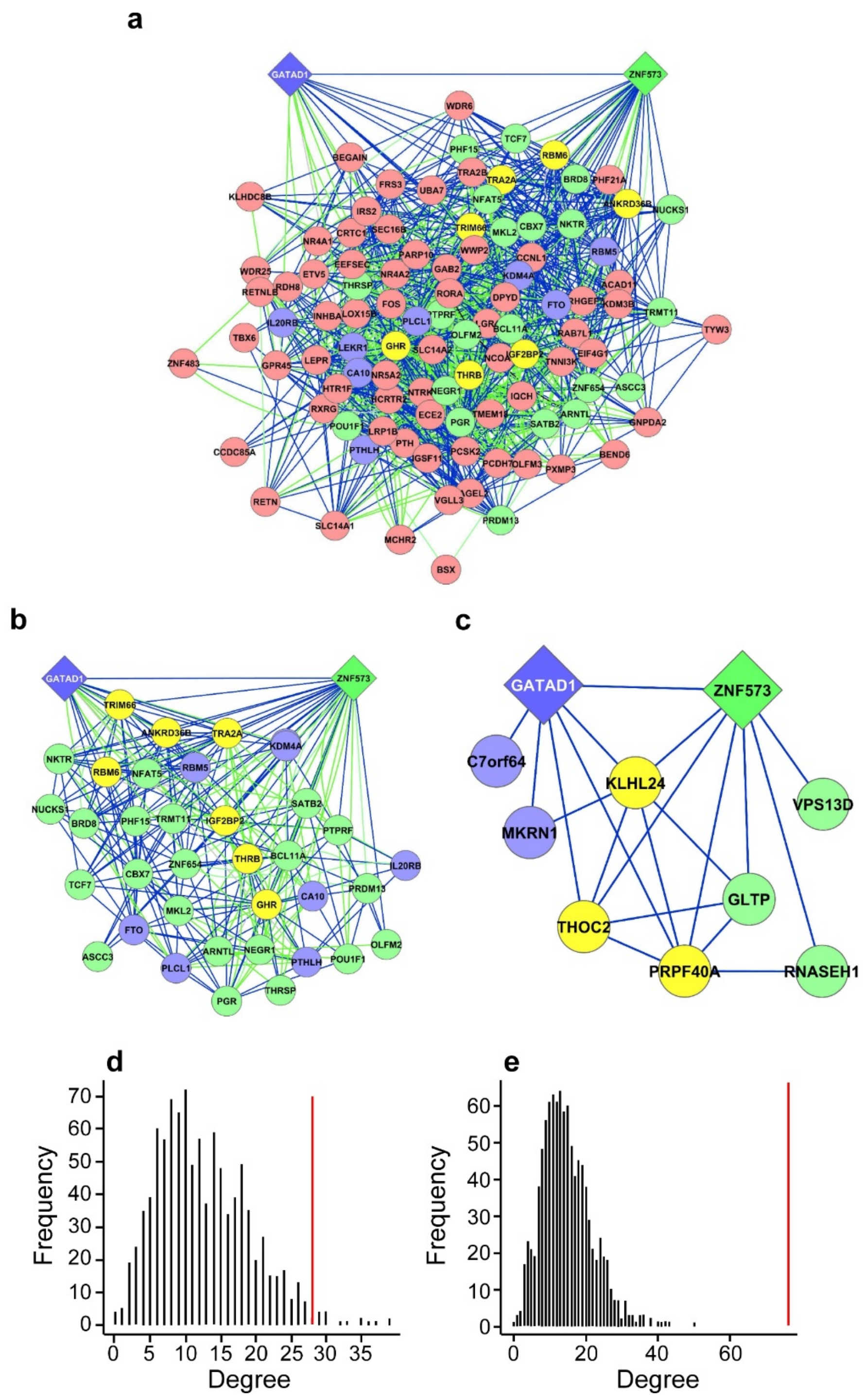


**Supplementary Figure 8: Interactions between *GATAD1* and *ZNF573* with genes identified by GWAS as associated with the age at menarche. a)** The interactions of *GATAD1* and

*ZNF573* with the complete set of menarche-associated genes from *Perry,J.R. et al* [2]. Blue nodes identify *GATAD1* and its first neighbors in the network, while *ZNF573* and its first neighbors are colored green. Pink genes are unconnected to *GATAD1* or *ZNF573*, while yellow genes are connected to both. Blue lines indicate a coexpression relationship, while green lines indicate a genetic interaction relationship. Redundant edges of the same type are not shown. **b)** Detail of the first-neighbor network of *GATAD1* and *ZNF573* from a. **c)** The first neighbor network of *GATAD1* and *ZNF573* from a randomly selected set of genes of equal size to the age of menarche-associated genes as indicated by GeneMANIA. **d, e)** Distribution of the connectivity of *GATAD1* (d) and *ZNF573* (e) in networks derived from 1000 randomly selected gene sets of equal size to the age of menarche-associated gene set. Connectivity of *GATAD1* and *ZNF573* to the menarche-associated gene set is indicated by the red lines.

**Supplementary Figure 9**

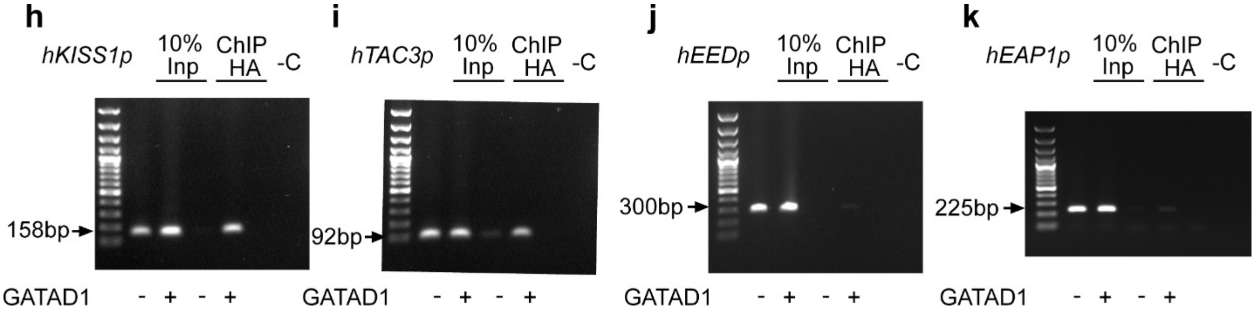


**Supplementary Figure 9: Uncropped Images of Figure 8**

**Supplementary Figure 10**

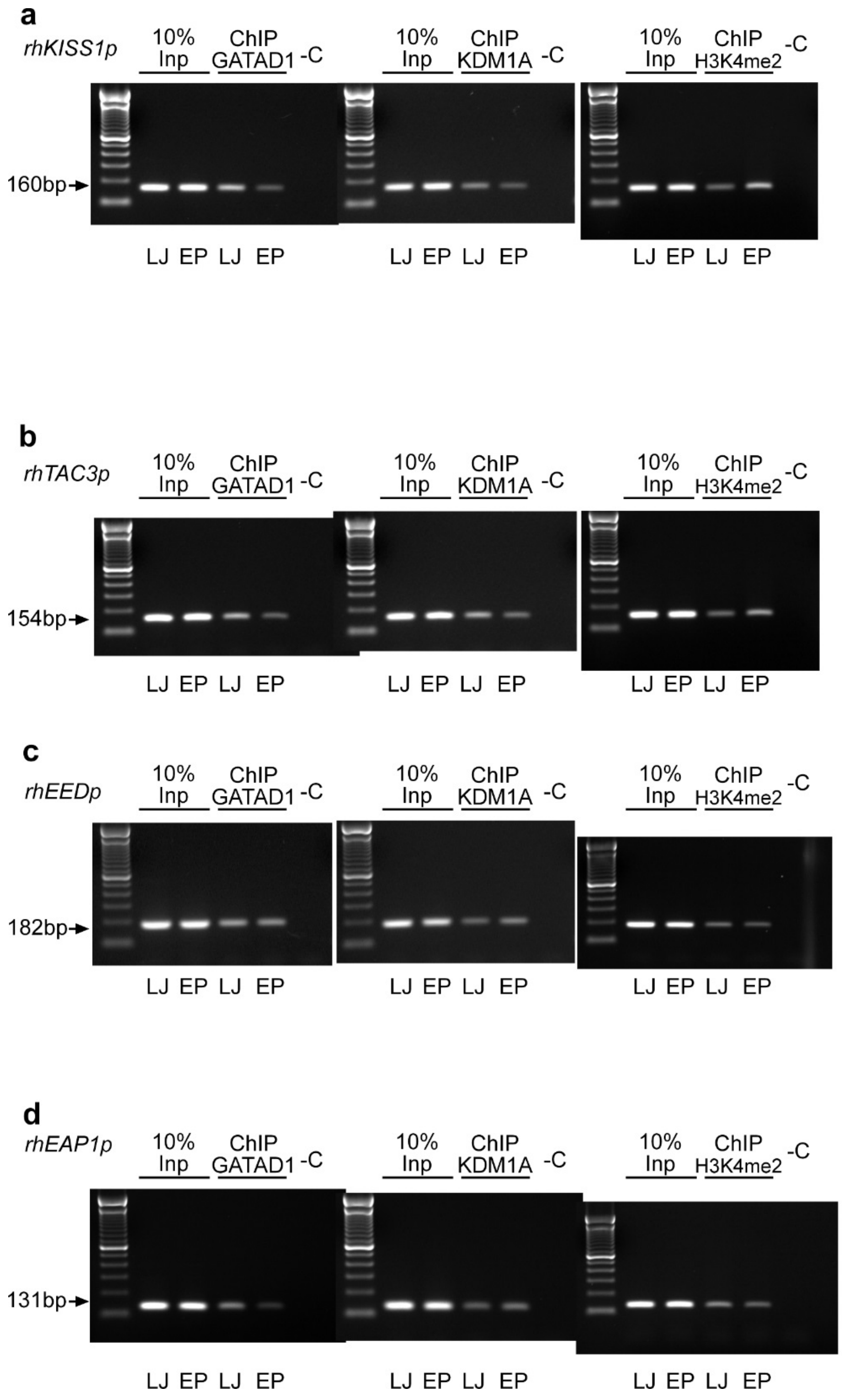


**Supplementary Figure 10: Uncropped Images of Figure 9**

**Supplementary Table 1**

| Target | Host | Source | Catalog # | Use |
|---|---|---|---|---|
| HA | Mouse | Covance | MMS-101R | WB/ChIP |
| HA | Rabbit | Abcam | ab9110 | WB/ChIP |
| Myc | Rabbit | Thermo | MA5-15125 | WB |
| GFP | Goat | Abcam | ab6673 | IHF |
| H3K4me3 | Rabbit | Active Motif | 39159 | ChIP |
| H3K4me2 | Rabbit | Abcam | ab7766 | ChIP |
| KDM1a | Rabbit | Bethyl Lab | A300-215A | WB/ChIP |
| GATAD1 | Mouse | Abcam | ab50816 | ChIP |
| Beta-Galactosidase | Rabbit | Cortex Biochem | CR7001RP2 | ChIP |
| Beta-Galactosidase | Mouse | Boehringer Mannheim | 1083 104 | ChIP |

**Supplementary Table 1:** Antibodies used in Western Blot, Immunohistofluorescence and ChIP assays.

**Supplementary References**


1. Heger,S. et al. Enhanced at puberty 1 (EAP1) is a new transcriptional regulator of the female neuroendocrine reproductive axis. J. Clin. Invest 117, 2145-2154 (2007).

2. Perry,J.R. et al. Parent-of-origin-specific allelic associations among 106 genomic loci for age at menarche. Nature 514, 92-96 (2014).
